# A Parameter Representing Missing Charge Should Be Considered when Calibrating Action Potential Models

**DOI:** 10.3389/fphys.2022.879035

**Published:** 2022-04-26

**Authors:** Yann-Stanislas H. M. Barral, Joseph G. Shuttleworth, Michael Clerx, Dominic G. Whittaker, Ken Wang, Liudmila Polonchuk, David J. Gavaghan, Gary R. Mirams

**Affiliations:** ^1^ Roche Pharma Research and Early Development, Pharmaceutical Sciences, Roche Innovation Center Basel, F. Hoffmann-La Roche Ltd., Basel, Switzerland; ^2^ Department of Computer Science, University of Oxford, Oxford, United Kingdom; ^3^ Centre for Mathematical Medicine and Biology, School of Mathematical Sciences, University of Nottingham, Nottingham, United Kingdom

**Keywords:** action potential, electrophysiology, mathematical model, conservation of charge, parameter fitting, calibration

## Abstract

Computational models of the electrical potential across a cell membrane are longstanding and vital tools in electrophysiology research and applications. These models describe how ionic currents, internal fluxes, and buffering interact to determine membrane voltage and form action potentials (APs). Although this relationship is usually expressed as a differential equation, previous studies have shown it can be rewritten in an algebraic form, allowing direct calculation of membrane voltage. Rewriting in this form requires the introduction of a new parameter, called Γ_0_ in this manuscript, which represents the net concentration of all charges that influence membrane voltage but are not considered in the model. Although several studies have examined the impact of Γ_0_ on long-term stability and drift in model predictions, there has been little examination of its effects on model predictions, particularly when a model is refit to new data. In this study, we illustrate how Γ_0_ affects important physiological properties such as action potential duration restitution, and examine the effects of (in)correctly specifying Γ_0_ during model calibration. We show that, although physiologically plausible, the range of concentrations used in popular models leads to orders of magnitude differences in Γ_0_, which can lead to very different model predictions. In model calibration, we find that using an incorrect value of Γ_0_ can lead to biased estimates of the inferred parameters, but that the predictive power of these models can be restored by fitting Γ_0_ as a separate parameter. These results show the value of making Γ_0_ explicit in model formulations, as it forces modellers and experimenters to consider the effects of uncertainty and potential discrepancy in initial concentrations upon model predictions.

## 1 Introduction

Since the seminal work by Hodgkin and Huxley (1952), mathematical models of electrophysiology have been developed for many different cell types, including neurons, cardiomyocytes, gastric smooth muscle cells, and many more (Noble, 1962; Dodge and Cooley, 1973; Corrias and Buist, 2007). Differences in ionic concentrations across cell membranes lead to a transmembrane voltage (*V*_m_). Its evolution over time is usually calculated in mathematical models by numerically integrating the effects of the ionic currents passing through the membrane. Since the late 90s, several authors have showed that *V*_m_ can also be computed directly from intra- and extracellular concentrations of charges, due to a conservation principle in the models (Guan et al., 1997; Varghese and Sell, 1997; Endresen et al., 2000; Hund et al., 2001; Jacquemet, 2007; Livshitz and Rudy, 2009; Pan et al., 2018). In this work, we investigate further the implications of using this second expression for *V*_m_ in terms of numerical stability, we highlight its impact on electrophysiological predictions, and we discuss the benefits to using this approach in model calibration.

First, in this section we present a brief overview of relevant work that leads to different ways of computing the voltage in AP models, based on a conservation of charge principle hidden in the equations, as well as how this conservation of charge relates to the steady state of the AP models. Section 2 then highlights how the accuracy of solutions is improved by the algebraic expression for voltage. In Section 3, we show that model outputs are sensitive to the net concentration of charge across the cell membrane, which varies because of high variability and/or uncertainty in initial concentrations. We finally show in Section 4 that Γ_0_, a parameter characterising the relationship between *V*_m_ and the intra- and extracellular concentrations of charges, can be inferred from experimental data to produce the desired steady-state behaviour of the AP model, despite being challenging to estimate experimentally.

In this study, we explore the consequences of writing *V*_m_ algebraically using the Ten Tusscher-Panfilov model of human ventricular cells (TTP06) (Ten Tusscher and Panfilov, 2006) and the CiPA version of the O’Hara-Rudy model by Dutta et al. (2017) (ORd-CiPA). Beyond these two models, our findings apply to any model tracking the intracellular concentrations of all charge-carriers, which make up the majority of modern electrophysiology models.

### 1.1 Membrane Voltage and Ionic Concentrations in AP Models

Major variables in AP models include *V*_m_, channel and pump/transporter state variables and, in later models, concentrations of ions, buffers, and signalling molecules. The relationship between these variables, grouped together in a vector **X**, is expressed as a system of ordinary differential equations (ODEs) of the form

$$\begin{array}{ll} \frac{dX}{dt} & =f\left(X\right),\\ X & =\left\{V_{\text{m}},C,g\right\}, \end{array}$$

where the vector function *f*(**X**) describes the rate of change of **X**, which can be subdivided into *V*_m_, the ionic concentrations **C** and all other variables **g**. The first equation in *f* is usually the one that defines the rate of change in *V*_m_, using an ideal capacitor equation:

$$\frac{dV_{\text{m}}}{dt}=-\frac{1}{C_{\text{m}}}\overset{N}{\underset{j=1}{\sum }}I_{j}\left(X\right),$$ (1)

where *C*_m_ is the membrane capacitance (usually in pF), and *I*_*j*_ are the *N* different ionic currents flowing across the cell membrane (in pA). Note that the currents depend non-linearly on voltage, concentration, and time, so that all the state variables are coupled together in a non-linear system.

The earliest AP models (e.g. Hodgkin and Huxley, 1952; Noble, 1962; McAllister et al., 1975) approximated intracellular concentrations as constants, arguing that the relatively small ionic currents would not alter concentrations significantly. This assumption holds well for the K^+^ and Na^+^ currents included in these models, which have relatively large internal concentrations which do not show significant variations during a single AP. In addition, simulating longer time spans during which these small changes could build up, was computationally infeasible at the time. But after the discovery of Ca^2+^ currents in the 60s, it was quickly realised that ${\left[{\text{Ca}}^{2+}\right]}_{\text{i}}$ could vary by orders of magnitude during a single AP, necessitating the inclusion of a time-varying ${\left[{\text{Ca}}^{2+}\right]}_{\text{i}}$ in models as early as the Beeler and Reuter (1977) model.

Later, DiFrancesco and Noble (1985) proposed a model where the current-induced changes in ${\left[{\text{Ca}}^{2+}\right]}_{\text{i}}$ , ${\left[K^{+}\right]}_{i}$ , and ${\left[{\text{Na}}^{+}\right]}_{\text{i}}$ are tracked over time, along with the extracellular concentration of K^+^ close to the cell membrane. This revolutionised the understanding of major features of cardiac electrophysiology, as reviewed by Dibb et al. (2015). Most subsequent AP models have retained the dynamic description for *intracellular* concentrations (although ${\left[K^{+}\right]}_{i}$ is sometimes held constant) and extended it with concentrations in intracellular compartments such as the sarcoplasmic reticulum (SR, e.g., Noble et al., 1991; Wilders et al., 1991; Luo and Rudy, 1994) and other species (e.g. chloride in Tomek et al., 2020). Variations in extracellular concentrations over the course of the action potential proved less popular but are still present e.g., in some models of atrial (Hilgemann and Noble, 1987; Lindblad et al., 1996; Nygren et al., 1998) and sino-atrial (Demir et al., 1994; Dokos et al., 1996; Lovell et al., 2004; Pohl et al., 2016) action potentials. Even though extracellular concentrations do vary in practice (e.g., under ischemic conditions), their variations due to ionic currents are often neglected in AP models because ions are constantly exchanged with the vascular buffer which limits their temporal variation in the extracellular space (Dokos et al., 1996) and reduces accumulation of ions in the extracellular space.

### 1.2 Algebraic Expressions for *V*_m_

A study by Varghese and Sell (1997) showed that models in which all membrane currents are assigned to a charge-carrying species, and in which the intracellular ionic concentrations vary accordingly, will implicitly satisfy a *conservation of charge* principle. As a result, *V*_m_ can be computed algebraically as a function of the concentrations, so that the ODE for *V*_m_ Eq. 1 is redundant. Applying the approach of Varghese & Sell to the Luo and Rudy (1994) model as an example, we obtain

$$V_{\text{m}}=\frac{V_{\text{i}}F}{C_{\text{m}}}\left({\left[{\text{Na}}^{+}\right]}_{\text{i}}+{\left[{\text{K}}^{+}\right]}_{\text{i}}+2{\left[{\text{Ca}}^{2+}\right]}_{i}+2\frac{V_{\text{JSR}}}{V_{\text{i}}}{\left[{\text{Ca}}^{2+}\right]}_{\text{JSR}}+2\frac{V_{\text{NSR}}}{V_{\text{i}}}{\left[{\text{Ca}}^{2+}\right]}_{\text{NSR}}\right)+\;V_{0},$$ (2)

where *V*_0_ is an integration constant (called *C*_0_ in the original publication), F is the Faraday constant, $V_{i}$ is the volume of the cytosol compartment of the cell, $V_{JSR}$ and $V_{NSR}$ are the volumes of the junctional (JSR) and network (NSR) sarcoplasmic reticulum compartments of the cell, respectively, and ${\left[{\text{Ca}}^{2+}\right]}_{\text{JSR}}$ and ${\left[{\text{Ca}}^{2+}\right]}_{\text{NSR}}$ are the concentrations of Ca^2+^ in these compartments. Hund et al. (2001) used a similar expression for *V*_m_ but moved the integration constant within the brackets, thereby turning it into a concentration instead of a voltage. Using *C*_0_ to represent the concentration, the two representations are related by $V_{0}=-\frac{V_{\text{i}}F}{C_{\text{m}}}C_{0}$ .

Endresen et al. (2000) proposed an expression very similar to that of Varghese and Sell but with a strong assumption: that all charges contributing to *V*_m_ are carried by K^+^, Na^+^, and Ca^2+^. This assumption leads to

$$V_{0}=-\frac{V_{\text{i}}F}{C_{\text{m}}}\left({\left[{\text{K}}^{+}\right]}_{\text{o}}+{\left[{\text{Na}}^{+}\right]}_{\text{o}}+2{\left[{\text{Ca}}^{2+}\right]}_{\text{o}}\right),$$ (3)

where [*X*]_o_ is the extracellular concentration of species *X*. In other words, *V*_m_ is simply proportional to the difference between total intracellular and extracellular concentrations of these three species. Endresen et al. acknowledged that their approach omitted anions, but justified this with the observation that the total concentrations of anions are approximately the same inside and outside the cell and that most currents are carried by cations. However, this framework needs to be extended for models which include Cl^−^, e.g., Hund and Rudy (2004); Grandi et al. (2010); Tomek et al. (2020): Eqs 2, 3 can be combined and generalised to any number of modelled species and compartments as follows

$$V_{\text{m}}=\frac{V_{\text{i}}F}{C_{\text{m}}}\left(\underset{A}{\sum }\underset{k}{\sum }z_{A}{\left[A\right]}_{\text{total},k}\frac{V_{k}}{V_{\text{i}}}-\underset{A}{\sum }z_{A}{\left[A\right]}_{\text{o}}\right),$$ (4)

where *A* represents each charged species in the model, *z*_*A*_ its valence, $V_{k}$ is the volume of the compartment *k* and the index *k* is over all intracellular compartments (e.g. compartment *k* = *i* corresponds to the cytosol). Equation 4 therefore accommodates further electrically charged species such as chloride, provided that the model keeps track of changes in their intracellular concentrations.

Note that the total concentration of any ion *A* is denoted here as [*A*]_total,*k*_. Some models include buffering of ions which alters free ionic concentrations, but as binding to buffers does not cause current flow over the membrane it should not change membrane voltage. So the [*A*]_total_ notation in Eq. 4 serves as a reminder that the total concentration carried by *A* is given by the sum of any buffered and free concentrations. For example, in many models ${\left[{\text{Ca}}^{2+}\right]}_{\text{total, i}}$ is not equal to ${\left[{\text{Ca}}^{2+}\right]}_{\text{i}}$ . This can make derivation of an algebraic-*V*_m_ form more complicated than in the examples above.

However, various other charge-carriers—ions, compounds and charged proteins—are known to be present at different concentrations on either side of the membrane, but are omitted from models. If these omitted charge carriers lead to a net transmembrane voltage, then an extra parameter is needed to account for the contribution of their charge imbalance to *V*_m_. For example, the Hund and Rudy (2004) dog action potential model includes Cl^−^ ions and an extra offset parameter would be needed to compensate the strong imbalance between intracellular ( ∼20 mM) and extracellular ( ∼100 mM) concentrations of Cl^−^, or there would be huge voltages using Eq. 4. In this model, chloride co-transporters change intracellular K^+^, Na^+^ and Cl^−^ concentrations but do not induce any ionic current or change voltage as they transport pairs of oppositely charged ions. The balanced effect of these co-transporters does not need special treatment in the equations above as long as both co-transported ionic species are accounted for.

We can modify Eq. 4 to explicitly allow for transmembrane imbalance of species that are not included in the model:

$$V_{\text{m}}=\frac{V_{\text{i}}F}{C_{\text{m}}}\left(\underset{A}{\sum }\underset{k}{\sum }z_{A}{\left[A\right]}_{\text{total},k}\frac{V_{k}}{V_{\text{i}}}-\underset{A}{\sum }z_{A}{\left[A\right]}_{\text{o}}\right)+\Delta V.$$ (5)

Here, Δ*V* corresponds to the transmembrane potential due to the difference in charge of *all* un-modelled species on either side of the membrane. As the contribution of these species to *V*_m_ is not modelled as varying, Δ*V* remains constant through the simulations. Equivalently, we can express the offset constant as a concentration that we denote Γ_0_:

$$V_{\text{m}}=\frac{V_{\text{i}}F}{C_{\text{m}}}\left(\underset{A}{\sum }\underset{k}{\sum }z_{A}{\left[A\right]}_{\text{tot},k}\frac{V_{k}}{V_{\text{i}}}-\underset{A}{\sum }z_{A}{\left[A\right]}_{\text{o}}+\Gamma_{0}\right),$$ (6)

where $\Gamma_{0}=V_{\text{i}}F\Delta V/C_{\text{m}}$ .

Expressing the offset as a concentration rather than voltage may help in assessing whether the values implicitly attributed to Γ_0_ by ODE models could be realistic. If positive, Γ_0_ could be interpreted as the net concentration of 1 + charged intracellular ions carried by species omitted in the model (or equivalently the net extracellular concentration of 1 − charged ions), and if negative it could be interpreted as a net intracellular concentration of 1 − charged omitted ions—but in reality it will reflect the sum of concentrations of a wide range of intra and extracellular un-modelled charged species. The smaller the magnitude of Γ_0_, the smaller the transmembrane imbalance of charge carried by un-modelled species. As a consequence, a value of Γ_0_ = 0 mM does not necessarily imply that no charge is missing in the model; but it does imply that any external missing charge is balanced exactly by an internal missing charge. Thus, the value of Γ_0_ must be interpreted in the light of which charged species are included in each model. Throughout this manuscript, we will use the Γ_0_ symbol to represent these missing charges, but the results hold equally well for its mathematically equivalent representation as voltage (Endresen et al., 2000), concentration of charge (Hund et al., 2001), or electrical charge (Jacquemet, 2007). Further detail on these expressions and their interpretation is provided in Supplementary Material Section S1-2.

A value for Γ_0_ can be found by substituting in the initial conditions for the concentrations and the initial value of *V*_m_ from the ODE formulation. This highlights an important point: models that express *V*_m_ in ODE form “hide” the value of this model parameter within their initial conditions. So when a set of initial conditions is chosen, perhaps arbitrarily from within the bounds of physiological realism, a hidden assumption is being made about the (im)balance of un-modelled charges in the cell. As we will show in this study, this net imbalance in un-modelled charge, captured by Γ_0_, is a key parameter in determining the behaviour of AP models.

### 1.3 Γ_0_ and Stable Behaviour

**FIGURE 1:**
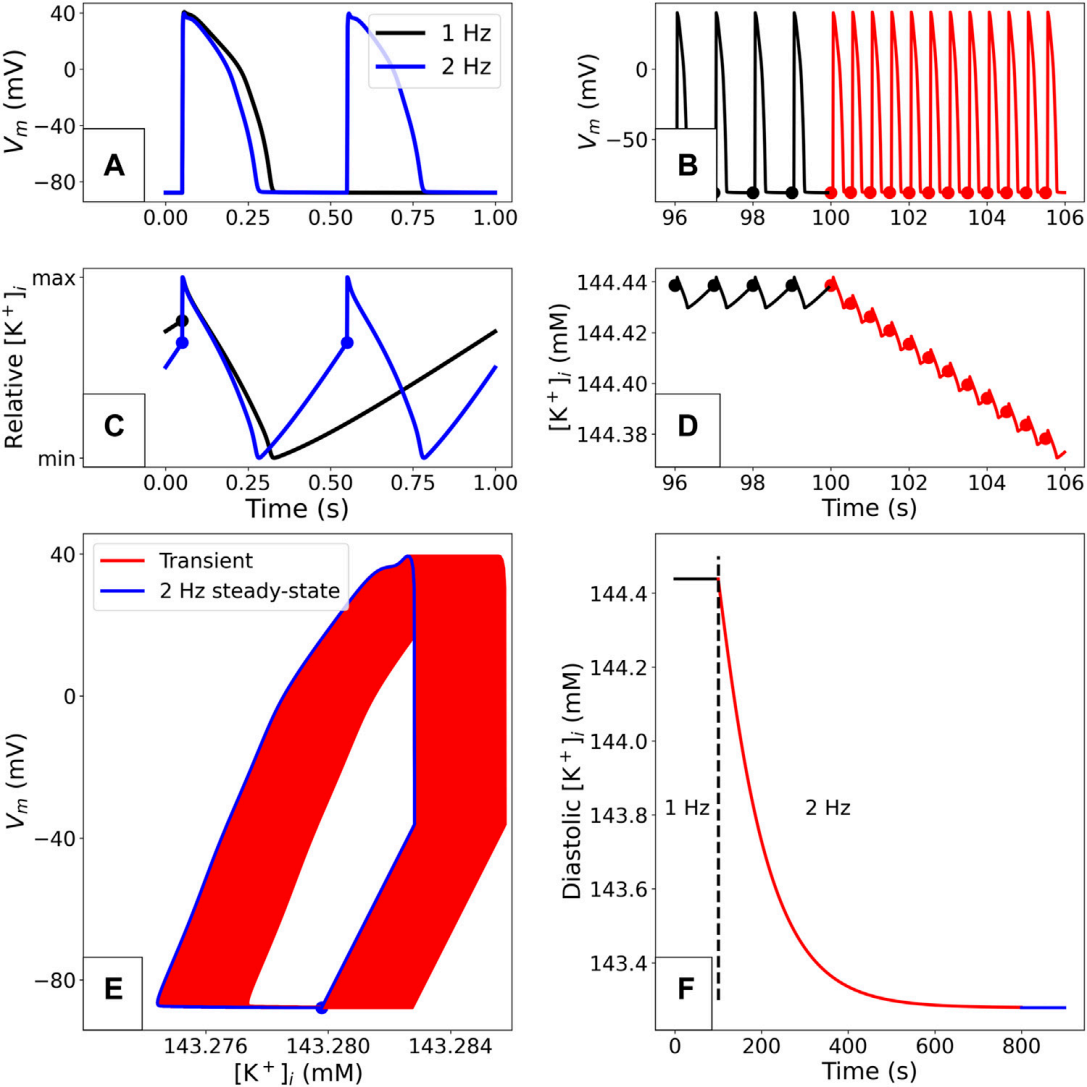
Example of a limit cycle in the O’Hara-Rudy CiPA 2017 model (Dutta et al., 2017), using the initial conditions from the published CellML model. The simulation methods are detailed in “Simulation”. **(A)**: Comparison of paced steady-state APs with 1 and 2 Hz pacing. **(B)**: Adaptation of the voltage profile when the pacing rate is suddenly changed from 1 to 2 Hz. The dots plotted on the traces correspond to the end of the diastolic phase in each AP. **(C)**: Comparison of periodic steady-state ${\left[K^{+}\right]}_{i}$ variations during the AP with 1 and 2 Hz pacing. The values are normalised for easier comparison. **(D)**: Adaptation of ${\left[K^{+}\right]}_{i}$ after the sudden change to 2 Hz shown in panel **(B)**. **(E)**: *V*_m_ and ${\left[K^{+}\right]}_{i}$ during the transient adaptation phase where the model converges towards its periodic steady state. Data is shown from the 500th pace onward. After a slow drift of ${\left[K^{+}\right]}_{i}$ over time, a limit cycle (in blue) is reached where the patterns from consecutive APs overlap. **(F)**: Evolution of diastolic intracellular potassium (measured at the time points denoted with dots in B and D) after a change in pacing rate. A limit cycle is reached after approximately 700 2 Hz paces.

In Figure 1 we show the stable behaviour of the O’Hara-Rudy CiPA model when paced for a long time at 1 Hz. The solution converges to a pattern under which all variables in the system take the same trajectory (to within numerical simulation tolerances) every time a stimulus is applied. The resulting periodic orbit in the state variable space (as shown in Figure 1E) is called a “stable limit cycle” in the study of dynamical systems, but is often referred to as a “steady state” for shorthand in electrophysiology modelling. Figure 1 also shows how a change in pacing to 2 Hz results in a transient shift to a new limit cycle. Similar transients to different limit cycles will also occur when other parameters in the model are changed (e.g., those representing maximal ion channel conductances being altered by drug block, or a change in extracellular concentrations). A model at a limit cycle has settled to a stable behaviour where each ionic concentration is in a dynamic equilibrium—any depletion/accumulation due to ions flowing down concentration gradients is restored before the next pace by pumps and exchangers (see Figure 1C).

Convergence to a stable limit cycle of the same period as the pacing (a “period-1” orbit) is not guaranteed: some models’ variables/concentrations may simply keep drifting (perhaps reaching unrealistic levels); exhibit more complex behaviour such as alternans (a stable “period-2” limit cycle in which we arrive back at the same state after two stimuli periods rather than one); or even chaotic behaviour (Qu, 2011). If pacing is stopped altogether, model variables may converge to stable values—a “stable steady state”. In models that exhibit automaticity, a limit cycle can be reached without any periodic forcing applied by a stimulus current. In this manuscript, we will use either “limit cycle” or “periodic steady state” when referring to stable limit cycles, and “quiescent steady state” when referring to stable steady states without any periodic forcing by a stimulus current.

Many published models do not exhibit a periodic steady state. Hund et al. (2001); Jacquemet (2007) showed that models where variables drift can often be ‘fixed’ to produce periodic steady states by ensuring that all currents through the membrane, including the stimulus current, are taken into account in the concentration updates, i.e. by ensuring that charge is conserved (as well as other conservation laws, see Pan et al., 2018).

Even when a model does have a periodic steady state, for any models where *V*_m_ is written as a redundant ODE, the charge represented by Γ_0_ is defined by the initial conditions. As a result, arbitrarily varying initial conditions in the presence of this redundant ODE alters the parameterisation of the model (changes the amount of charge in the system), and any quiescent steady states or limit cycles can alter accordingly. Or in other words, when a redundant ODE is included there can be no unique periodic steady state, it will vary depending on the initial conditions. Conversely, when the redundant ODE is removed there is often a unique stable limit cycle or quiescent steady state; that is, the same quiescent steady state or limit cycle is reached for *any* initial conditions.

Some authors such as Livshitz and Rudy (2009) have gone a step further, and suggested that uniqueness of limit cycles/quiescent steady states is guaranteed once conservation of charge is met. An analysis by Jacquemet (2007), however, shows that more than one stable quiescent steady state can exist for a charge-conserving model with a given value of Γ_0_. Examining the atrial model by Nygren et al. (1998), Jacquemet found that for some values of Γ_0_ the model had a stable steady state where *V*_m_ is polarised at rest (−60 to −90 mV), a stable steady state where the cell is depolarised to about −30 mV, and an unstable periodic steady state where the model displays automaticity. In the course of this study we also found examples of more than one stable limit cycle in other analytic-*V*_m_ models, which are discussed below.

Although undoubtedly important for reproducible modelling, it is reasonable to question the physiological relevance of quiescent steady states and limit cycles. Convergence to a perfect limit cycle seems unlikely to occur in real cells, as channel activity and other chemical processes are inherently stochastic and will perturb each orbit differently. The idea of a limit cycle, however, overlaps well with biological concepts of homeostasis and robustness. Even though the cell’s environment is constantly altering to some degree, it would be ideal for a cell to exist in close proximity to a stable limit cycle such that small stochastic perturbations converge back to the same behaviour—at least while energetic demands are met.

## 2 Impact of the Algebraic Voltage Formulation on Numerical Solutions

### 2.1 Models and Simulation

CellML files for the TTP06 and ORd-CiPA models were obtained from the Physiome Model Repository (Yu et al., 2011). The TTP06 model has epi-, endo- and mid-myocardial variants; where not stated otherwise we used the epicardial variant in this study. The units in the obtained CellML files for TTP06 had to be corrected before the algebraic-*V*_m_ form could be applied, as described in Supplementary Material Section S1.1. The algebraic-*V*_m_ forms of the TTP06 and ORd-CiPA models were derived, and model variants that employ this form were created for comparison with the original derivative-*V*_m_ form. A detailed overview of the conversion of a model to its algebraic-*V*_m_ form is given in Supplementary Material Section S1.3, along with a guide to performing this translation in other models.

Simulations were performed using Myokit (Clerx et al., 2016) which imported the CellML models, and using solver tolerances stated in the section below. Unless stated otherwise, figures were created after 2000 pre-pacing stimuli at a frequency of 1 Hz. In the TTP06 model, the stimulus current was modelled as a K^+^ current of amplitude −52 A/F lasting 0.5 ms. In the ORd-CiPA model, the stimulus current was also attributed to K^+^ ions and its amplitude was set at −50 A/F and its duration at 1 ms.

All code used for this article is publicly available and open source (see *Data Availability* at the end of the article).

### 2.2 Accuracy of Solutions

**FIGURE 2:**
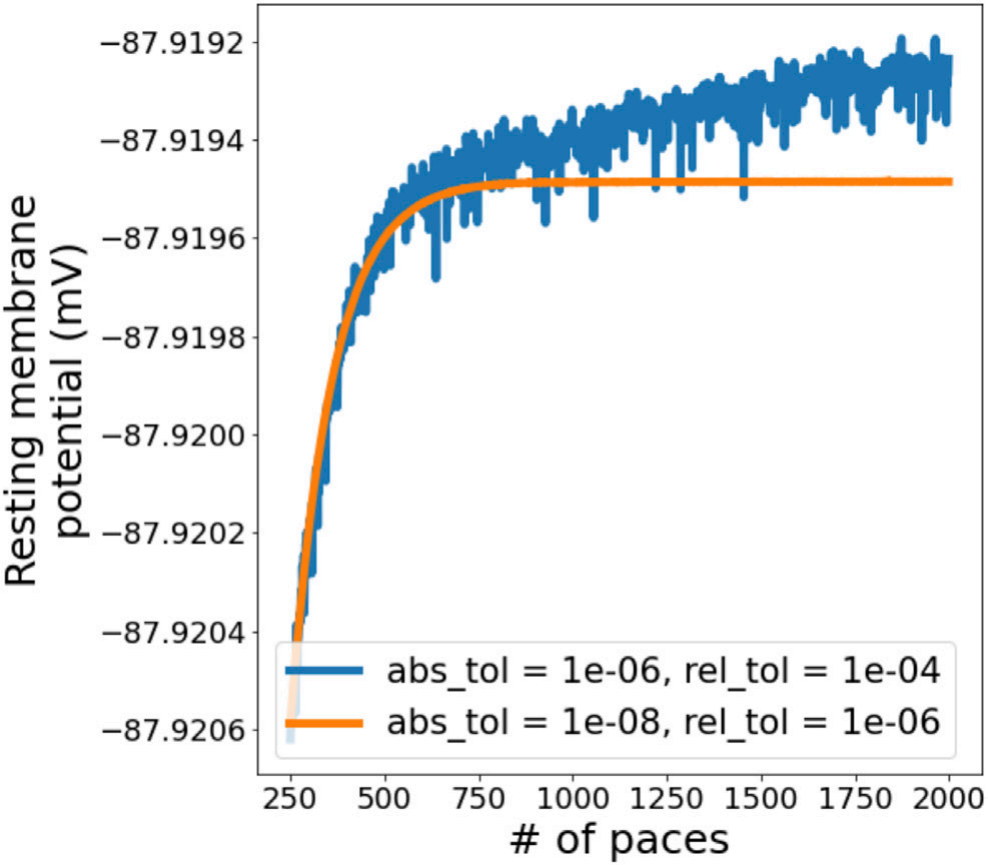
Evolution of resting membrane potential (RMP) in a simulation with the derivative-*V*_m_ ORd-CiPA model, starting from the published initial conditions. 2000 paces were simulated, we are showing paces 250 onwards to examine the behaviour close to periodic steady state. A slight drift is observed when using a coarse solver tolerance, but this disappears when tolerances are tightened.

Simulations in Myokit are performed using the CVODES software package (Hindmarsh et al., 2005) to numerically integrate the differential equations. CVODES has two “tolerance” settings that control the accuracy of the numerical solutions (Cohen et al., 1996). To visualise the influence of solver tolerance on AP simulations and find suitable tolerances to use in this study, simulations were run for 2000 paces with the ORd-CiPA model in its derivative-*V*_m_ form, using a coarse setting (10^–6^ and 10^–4^ for absolute and relative tolerance, respectively) and a fine setting (10^–8^ and 10^–6^). The resting membrane potential (RMP) was measured as the *V*_m_ 1 ms before application of the stimulus, and plotted for the final 1750 paces in Figure 2.

As expected, using coarse tolerances results in (a small) numerical error in the solution, but the figure also shows a slight drift in *V*_m_, even after 1,000 paces. When tightening the solver tolerance, the numerical noise is significantly reduced, and *V*_m_ stabilises after around 700 paces. The other state variables show a similar pattern, as can be seen for ${\left[K^{+}\right]}_{i}$ in Supplementary Material Section S1.4.

**FIGURE 3:**
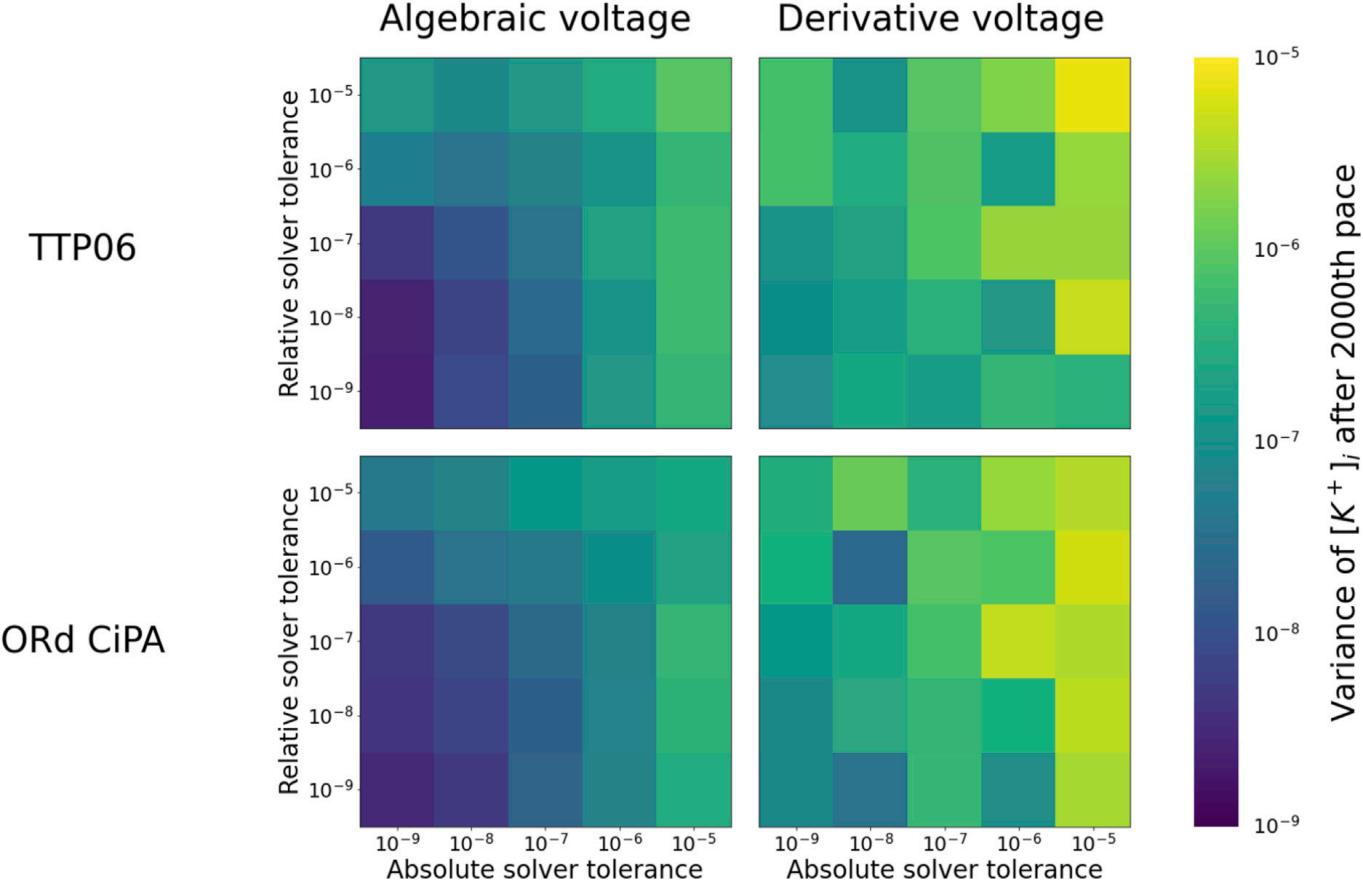
Numerical stability of ${\left[K^{+}\right]}_{i}$ in the TTP06 and ORd-CiPA models, comparing the derivative and algebraic-*V*_m_ forms. The colour map corresponds to the standard deviation of ${\left[K^{+}\right]}_{i}$ between the 2000th and 3000th pace. The darker the map, the lower the variance, and the more stable the simulation.

To further investigate the long term stability of the solutions, 3,000 paces were simulated with the ORd-CiPA and TTP06 models, in both the derivative and the algebraic-*V*_m_ forms. Since, with fine tolerances, the system had stabilised after 2000 paces (see Figure 2), the variation in the state variables after 2000 paces could safely be attributed to numerical error and not to electrophysiological phenomena. We quantified this variation by measuring the standard deviation in the final 1,000 paces in ${\left[K^{+}\right]}_{i}$ (the state variable that had the highest absolute value and largest variations over successive paces, see Supplementary Material Section S1.4). This standard deviation was evaluated for several solver tolerances, in both the derivative and algebraic-*V*_m_ forms of the models, and plotted in Figure 3 to create a “map of stability”.

For both models, numerical solutions appear less stable when using the derivative-*V*_m_ form Eq. 1. We believe this is because the intracellular ionic concentrations and *V*_m_ are updated without the numerical method having any knowledge of Γ_0_. This can lead to numerical errors that break conservation of charge, effectively introducing variations in Γ_0_, and allowing the periodic steady state of the system to change. By contrast, when explicitly incorporating the algebraic constraint on *V*_m_ (Eq. 6) and fixing Γ_0_, conservation of charge is guaranteed, so that the periodic steady state stays the same and the stability of the solution is improved.

For the remainder of this manuscript, we therefore used the algebraic-*V*_m_ form and absolute and relative solver tolerances of 10^–8^ and 10^–6^, respectively.

### 2.3 Computation Time

We also investigated whether computation time was affected by switching to the algebraic-*V*_m_ form of the model. One might have expected an improvement in simulation time due to a smaller and better conditioned system with the redundant ODE removed (avoiding a singular Jacobian as Varghese and Sell (1997) suggested), but there was no significant (if any) change in computation time, see Supplementary Figure S5 in the Supplementary Material.

## 3 Physiological Impact of Γ_0_

### 3.1 Γ_0_, ${\left[K^{+}\right]}_{i}$ and ${\left[{\text{Na}}^{+}\right]}_{\text{i}}$ in Human Ventricular AP Models

The algebraic-*V*_m_ form of the model (Eq. 6) gives the voltage in terms of the total intra- and extra-cellular ionic concentrations. The impact of variations in these parameters and variables across ventricular models was investigated by computing Γ_0_ for several literature models using the published initial conditions. This work could be carried out only for models which obey the conservation of charge principle. The results are shown in Table 1 which reports Γ_0_ (Eq. 6), the corresponding *C*_0_ as defined by Endresen et al., and the corresponding voltage offset Δ*V* for each of the investigated models.

**TABLE 1:** The integration constant for a range of human AP models, written as *C*_0_ (Hund et al., 2001)—see Section 1.2—, net un-modelled species concentration Γ_0_ Eq. 6, and voltage offset Δ*V* Eq. 5. The Trovato et al. (2020) and Stewart et al. (2009) models are Purkinje fibre models, while the remaining models represent ventricular cells.

Model	*C*_0_ (mM)	Γ_0_ (mM)	Δ*V* (mV)	Included ions
Trovato et al. (2020)	195.3377	−46.3377	−1.0605 × 10^6^	K^+^, Na^+^, Ca^2+^
Stewart et al. (2009)	147.2641	2.1359	1.8273 × 10^4^	K^+^, Na^+^, Ca^2+^
Ten Tusscher et al. (2004) Epi/Endo	150.5207	−1.1207	−9.5878 × 10^3^	K^+^, Na^+^, Ca^2+^
Ten Tusscher and Panfilov (2006) Epi	147.2683	2.1317	1.8237 × 10^4^	K^+^, Na^+^, Ca^2+^
Ten Tusscher and Panfilov (2006) Endo	150.5427	−1.1427	−9.776 × 10^3^	K^+^, Na^+^, Ca^2+^
Iyer et al. (2004)	135.7501	10.2499	1.6659, ×, 10^5^	K^+^, Na^+^, Ca^2+^
O’Hara et al. (2011) Endo	156.8010	−7.8010	−1.2680, ×, 10^5^	K^+^, Na^+^, Ca^2+^
O’Hara et al. (2011) Epi	156.8022	−7.8022	−1.2682 × 10^5^	K^+^, Na^+^, Ca^2+^
Dutta et al. (2017) (ORd-CiPA) Endo	156.8011	−7.8011	−1.2680 × 10^5^	K^+^, Na^+^, Ca^2+^
Tomek et al. (2020) Epi	135.7563	−137.1563	−2.2294 × 10^6^	K^+^, Na^+^, Ca^2+^, Cl^−^
Tomek et al. (2020) Endo	135.7555	−137.1555	−2.2294 × 10^6^	K^+^, Na^+^, Ca^2+^, Cl^−^

These parameters contain information about the difference between the un-modelled intra- and extracellular charged species (e.g. H^+^, Mg^2+^, cations, phosphates, proteins). In the TTP06 Epi model, for example, the intra- and extra-cellular charges of these missing species are responsible for a voltage offset of 18.2 V. In the ORd-CiPA model, the voltage offset is of −126.8 V.

The Tomek et al. (2020) model (an update of the 2019 version to conserve charge) has a very high Γ_0_ constant due to the inclusion of chloride ions, for which there is a very large difference between intra- and extracellular concentrations. In the Ten Tusscher et al. (2004) model, the epicardial and endocardial versions were assumed to have the same initial conditions, so their missing charge concentrations are the same. The 2006 epi/endo variants of the Ten Tusscher model (Ten Tusscher and Panfilov, 2006) have minor differences in the initial conditions and buffered Ca^2+^ concentrations. As a result, there are slight differences in Γ_0_ between the various versions of the Ten Tusscher et al. model.

It remains to be seen whether the Γ_0_ value (net concentration of un-modelled charge) is biologically as variable as the values it has been implicitly assigned within models, or whether this simply reflects lack of information on real concentrations and subsequent uncertainty in what initial conditions should be used.

Comparing the magnitudes of Γ_0_ and Δ*V* in Table 1 shows that a 20 mV variation one might observe in resting potential between models corresponds to Γ_0_ variations of approximately 0.002  mM, much smaller than the variation in the offset constants between models. So what we observe is not influenced much by the precise value of the initial condition for the RMP (this is the same reason initial gating variable values have negligible effects) but instead by how the various possible initial concentrations cause longer term system behaviour to change *via* altered Nernst potential (or GHK flux equations) and resulting currents, as well as any explicit concentration-dependence in gating kinetics. So the impact of initial RMP on Γ_0_ can be neglected in comparison to that of initial concentrations (RMP is also much easier to measure to within a few millivolts in experiments). As a consequence, variation of the initial voltage used to compute Γ_0_ from Eq. 6 was neglected in this study and the initial voltage as published in the original models was used to compute Γ_0_ in simulations of the sections below.

### 3.2 Γ_0_ and Ranges of K^+^ and Na^+^

In this section, we estimate the variability of Γ_0_ from literature and observe how this variability might impact the AP predicted by the model. The values that can be taken by Γ_0_ are, for a large part, dictated by the uncertainty in intracellular concentrations in intact myocytes. Extracellular concentrations are fixed parameters in most AP models that are more reliably estimated (at least in *in vitro* experiments); we therefore investigate the effect of only the initial conditions of intracellular state variables on long-term model behaviour.

**FIGURE 4:**
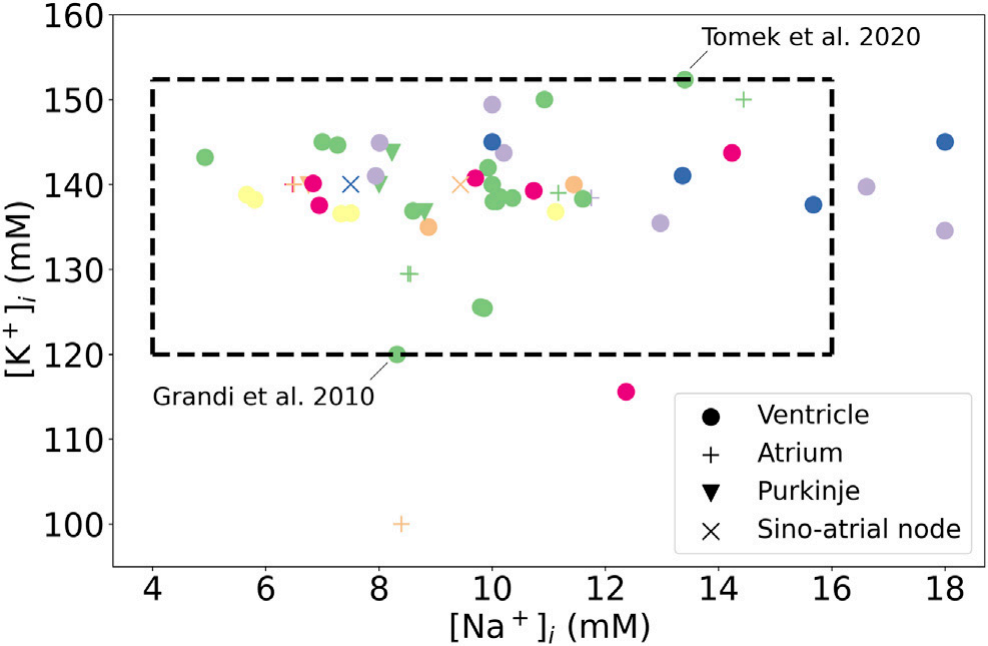
Initial concentrations published for cardiac AP models, for a range of species and tissues. Green: human, Purple: canine, Orange: rabbit, Yellow: Guinea pig, Blue: mammalian, Pink: murine. The dotted box highlights the extreme values of intracellular concentrations, estimated from the work of Bers et al. (2003) for Na^+^ and from the Grandi et al. (2010) and the Tomek et al. (2020) models for K^+^.

A literature search was carried out to find the range of intracellular K^+^ and Na^+^ concentrations observed experimentally in human cardiomyocytes and/or used in simulations. The contribution of Ca^2+^ to total intracellular charge at the end of the resting phase of the AP is much smaller, so its variation can be neglected compared to K^+^ and Na^+^, and Γ_0_ variation between the models is mainly due to different intra- and extra-cellular K^+^ and Na^+^. The concentrations of ${\left[K^{+}\right]}_{i}$ and ${\left[{\text{Na}}^{+}\right]}_{\text{i}}$ used in previous cardiac AP models are reported in Figure 4, for a range of tissues and species based on the annotated CellML models at https://github.com/Chaste/cellml that were studied in Cooper et al. (2015).

In human ventricular cardiomyocytes the intracellular sodium concentration ( ${\left[{\text{Na}}^{+}\right]}_{\text{i}}$ ) was found to range experimentally from 4 to 16 mM (Bers et al., 2003). Fry et al. (1986) determined experimentally that the intracellular potassium concentration ( ${\left[K^{+}\right]}_{i}$ ) is 113 ± 6 mM in rat cardiomyocytes. We did not find direct experimental measurements of ${\left[K^{+}\right]}_{i}$ in ventricular human cardiomyocytes in the literature. Also, experimental measurements of intracellular ionic concentrations in intact cardiomyocytes were all performed in the quiescent configuration. We therefore used initial values for ${\left[K^{+}\right]}_{i}$ from human ventricular AP models as a measure of uncertainty in ${\left[K^{+}\right]}_{i}$ , which ranged from 120 mM in the Grandi et al. (2010) model to 152 mM in the Tomek et al. (2020) model. With these estimated ranges for ${\left[K^{+}\right]}_{i}$ and ${\left[{\text{Na}}^{+}\right]}_{\text{i}}$ , the range for their sum varies by 44 mM. Such uncertainty in intracellular concentrations produces the high variability of Γ_0_ between models that is observed in Table 1.

The extreme K^+^ and Na^+^ concentrations from Figure 4 were used to initialise ${\left[K^{+}\right]}_{i}$ and ${\left[{\text{Na}}^{+}\right]}_{\text{i}}$ in simulations to observe the effect of such variations on the limit cycle AP. The K^+^ concentration was initialised to 120 mM and to 152 mM in the two models, whilst the initial Na^+^ concentration was initialised to 4 mM and to 16 mM, respectively. Γ_0_ was computed from Eq. 6 for these intracellular concentrations and initial voltage set to its published value (−84.9 mV for the TTP06 model, − 88 mV for the ORd-CiPA model). The high total concentration of intracellular ions yielded Γ_0_ = −20.4 mM and Γ_0_ = −24.4 mM in the TTP06 and the ORd-CiPA models, respectively. The low total concentration of intracellular ions yielded Γ_0_ = 23.6 mM and Γ_0_ = 20.9 mM in the TTP06 and the ORd-CiPA models, respectively.

In simulations in sections below where the value of Γ_0_ is imposed by the user, the initial intracellular concentrations must be changed to satisfy the algebraic constraint of Eq. 6 and leave the initial voltage unchanged. Otherwise, the high variations of Γ_0_ reported in Table 1 would lead to voltage offsets of up to several kilovolts. The intracellular concentration of K^+^ was therefore adjusted with Eq. 6 so that the initial voltage remains untouched and consistent with the required value of Γ_0_. Alternatively, Na^+^ could be adjusted; but the degree of variation of Γ_0_ could lead to negative values of ${\left[{\text{Na}}^{+}\right]}_{i}$ so we adjust K^+^ instead.

The ORd-CiPA model has extra ionic variables compared to the TTP06 model: variables were added for the concentrations of sodium and potassium in the subspace domain, denoted by [Na^+^]_SS_ and [K^+^]_SS_. At the limit cycle, the difference between diastolic concentrations of ions in the subspace and in the intracellular compartment were observed to be smaller than 0.1 mM, even when initial conditions were set to very different values (results not shown). Furthermore, there is no physical structure delimiting the subspace from the bulk intracellular space. Thus, K^+^ and Na^+^ concentrations in the subspace are very close to concentrations in the main intracellular compartments at the end of the resting phase of the AP, i.e., when state variables are initialised in simulations. To avoid introducing big differentials in K^+^ and Na^+^ concentrations between the subspace and the bulk cytosol compartment in simulations where the user introduced changes to initial conditions for ${\left[K^{+}\right]}_{i}$ and ${\left[{\text{Na}}^{+}\right]}_{\text{i}}$ , the initial conditions of [Na^+^]_SS_ and [K^+^]_SS_ were set to the same values as ${\left[{\text{Na}}^{+}\right]}_{\text{i}}$ and ${\left[K^{+}\right]}_{i}$ respectively.

**FIGURE 5:**
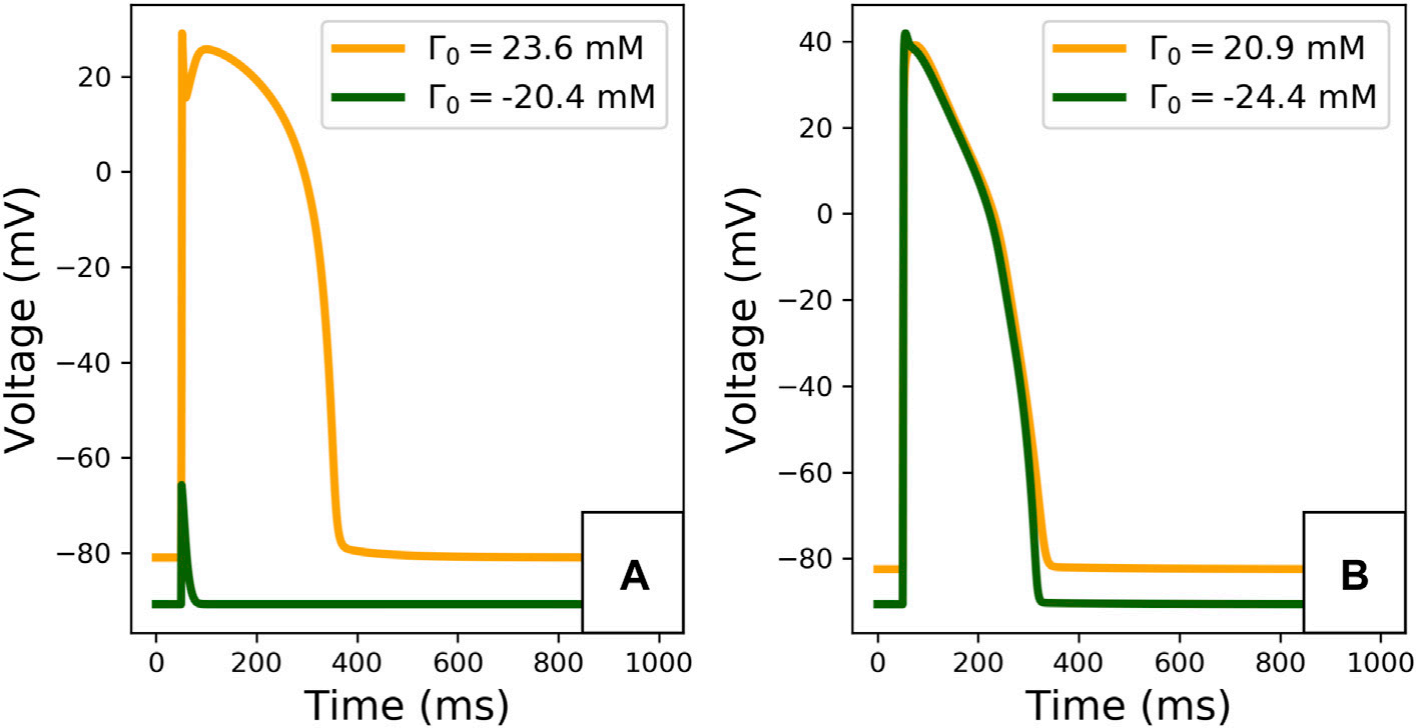
Limit cycle APs for extreme initial conditions for the TTP06 model **(A)** and for the ORd-CiPA model **(B)**. Extreme Γ_0_ values covering approximately 44 mM are computed from the extreme ${\left[K^{+}\right]}_{i}$ and ${\left[{\text{Na}}^{+}\right]}_{\text{i}}$ observed in human ventricular models, as reported in Figure 4.

The limit cycle APs, observed after 2,000 paces, are plotted in Figure 5. The difference in Γ_0_ induces important changes in the limit cycle AP, especially for the TTP06 model. For instance, the TTP06 model does not have a physiological AP when simulated with a very low Γ_0_ value, the cell does not depolarise. In the ORd-CiPA model, the RMP is particularly impacted, decreasing from −82 mV for Γ_0_ = −24.4 mM to −88 mV for Γ_0_ = 20.9 mM. This shows that Γ_0_ variations have a strong impact on the model output, which is investigated further below.

### 3.3 Effect of Γ_0_ on Steady States

Several authors have asserted that Γ_0_ (or its equivalents from the literature) defines the steady states of various models, both under paced and unpaced conditions (Hund et al., 2001; Jacquemet, 2007; Livshitz and Rudy, 2009; Pan et al., 2018). Here we investigate the steady states and limit cycles reached by the TTP06 and ORd-CiPA models for initial conditions that sample the range of physiologically-plausible Γ_0_ values (Section 3.2).

The range of experimental concentrations determined in the previous section was sampled at 10 linearly spaced Γ_0_ values. For each Γ_0_ value, the ${\left[{\text{Na}}^{+}\right]}_{\text{i}}$ range was sampled linearly at 10 points. The initial ${\left[{\text{Ca}}^{2+}\right]}_{\text{i}}$ was taken to range from 0.5 to 1.5 times its originally published value, also with 10 sampling points, giving a total of 100 samples for each Γ_0_ value. The remaining Ca^2+^ concentrations were initialised to a random value ranging from 0.5 to 1.5 times their published initial value. The initial value for ${\left[K^{+}\right]}_{i}$ was computed using Eq. 6 to match with the initial voltage of the published model. Due to the linear relationship between the ionic concentrations in Eq. 6, a hyperplane in the state variable space can be associated to each Γ_0_ value. The initial values of the remaining state variables (gating variables) were taken randomly within the range 0–1, and the sum of the Markov states in the I_Kr_ compartment of the ORd-CiPA model was maintained equal to 1. The quiescent steady state was reached after 4000 s without pacing and the limit cycle was recorded after 2000 s of steady 1 Hz pacing, and the values of the state variables at the end of the diastole were recorded.

**FIGURE 6:**
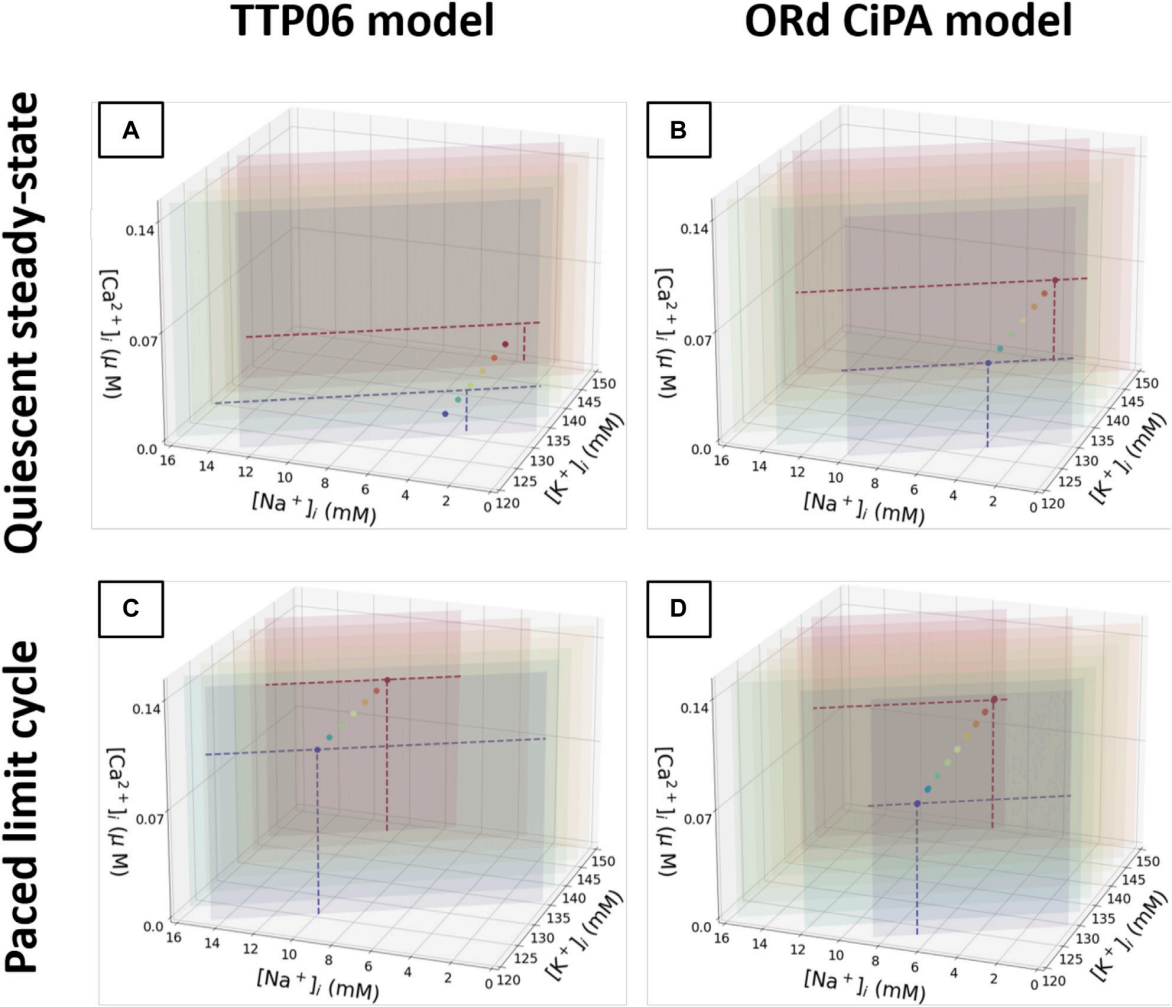
Plot of the quiescent steady state and limit cycle values for ${\left[{\text{Na}}^{+}\right]}_{i}$ , ${\left[K^{+}\right]}_{i}$ and ${\left[{\text{Ca}}^{2+}\right]}_{\text{i}}$ . **(A)**: TTP06 model at a quiescent steady state. **(B)**: ORd-CiPA model at a quiescent steady state. **(C)**: TTP06 model in a limit cycle. **(D)**: ORd-CiPA model in a limit cycle. Each plane has initial conditions satisfying Eq. 6 with the same fixed Γ_0_ value. 100 combinations of initial conditions are sampled from each plane to cover the physiological range of concentrations. These initial conditions are used in simulations to reach the (top row) quiescent steady state and the (bottom row) paced limit cycle. The steady state and limit cycle concentrations are plotted as points (with dashed projections along the associated Γ_0_ plane), with the colour matching the plane from which the initial conditions were sampled. For clarity, the planes for which the quiescent steady state is out of the range reported in Section 3.2, are not shown.

The quiescent steady state and the 1 Hz limit cycle diastolic intracellular concentrations are shown in Figure 6. For each Γ_0_ value, all the simulations converged to the same quiescent or periodic steady state. The steady states that can be reached by the models for the various Γ_0_ values align on these plots.

Note how some of the points in Figure 6A appear to move outside the Γ_0_ plane. Only ${\left[K^{+}\right]}_{i}$ , ${\left[{\text{Na}}^{+}\right]}_{\text{i}}$ , and ${\left[{\text{Ca}}^{2+}\right]}_{\text{i}}$ are plotted to allow a 3D visualisation of the quiescent steady states and limit cycles. Thus, major changes in other concentrations, which are not plotted in the figure, shift the steady states. Although the steady state variables appear outside of the initial Γ_0_ plane in this lower dimensional representation, Γ_0_ was correctly preserved throughout the simulations.

For both models, regardless of the initial conditions used for the state variables, a unique quiescent steady state and a unique 1 Hz limit cycle were observed for each value of Γ_0_. Thus, the solution of the model under quiescence and for prolonged regular pacing is defined by the value of Γ_0_. This observation is consistent with the studies mentioned previously, with constants equivalent to Γ_0_. As a conclusion, Γ_0_ can be used as a single model parameter to summarise the intracellular concentrations in these models at these pacing conditions and parameter values. Moreover, the initial conditions for the gating variables did not impact the limit cycle or steady-state outputs, so their initial conditions were not altered in further simulations. When calibrating an AP model based on its limit cycle or steady state outputs, it appears sufficient to establish the correct value of Γ_0_, regardless of how K^+^, Na^+^ and Ca^2+^ concentrations and gating variables are individually initialised as long as they remain physiologically plausible. Thus, when exploring values of Γ_0_ in a derivative-*V*_m_ model the changes could be attributed to a single intracellular concentration (K^+^ for example) without loss of generality.

### 3.4 Model Predictions Are Sensitive to Γ_0_

The influence of Γ_0_ on the limit cycle outputs and on the APD restitution portrait was evaluated in the TTP06 and ORd-CiPA models. The models’ outputs were recorded with Γ_0_ values varying by 30 mM. Intracellular concentrations were initialised so that Eq. 6 is satisfied with the initial voltage set to its published value. The state variables other than intracellular concentrations were initialised to their originally published initial values. 2000 paces were simulated to approach the limit cycle. The inward rectifier potassium current (I_K1_) and the sodium potassium exchanger current (I_NaK_), the currents which showed the highest sensitivity to Γ_0_ change, were recorded at 1 Hz pacing, together with *V*_m_.

**FIGURE 7:**
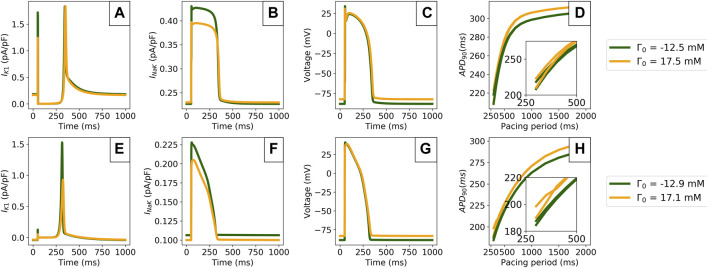
Comparison of model predictions in the periodic steady state outputs for the extreme values of Γ_0_ computed from Section 3.2. Data is shown for the TTP06 (top row) and ORd-CiPA models (bottom row). **(A,E)**: I_K1_ current. **(B,F)**: Sodium-Potassium exchanger (I_NaK_) current. **(C,G)**: AP. **(D,H)**: Limit cycle restitution portraits showing APD_90_ variation with the pacing period. The insets show pacing cycle lengths of 500 ms and shorter.

The AP duration restitution portrait at limit cycle was investigated using the Cardiac Electrophysiology Web Lab (https://chaste.cs.ox.ac.uk/WebLab) (Cooper et al., 2016; Daly et al., 2018). There, the models were loaded as CellML files, using the public protocol “Steady State Restitution”. In this protocol, 2000 paces are applied (bringing models close to their limit cycles) at various pacing periods ranging from 250 to 2000 ms. Two consecutive APs are then recorded, and their APD_90_s measured. The limit cycle outputs at 1 Hz and the restitution plots are shown in Figure 7.

Γ_0_ variations impacted the I_K1_ current particularly strongly in both models, with faster I_K1_ activation kinetics for lower Γ_0_ values, see Figures 7A,E. In addition, peak I_K1_ is decreased by 45% when increasing Γ_0_ by 30 mM in the ORd-CiPA model. I_NaK_ is also shown to be sensitive to Γ_0_, see Figures 7B,F. When using a low Γ_0_ value, I_NaK_ is reduced by approximately 15% in both the TTP06 and the ORd-CiPA models. The consequences for the simulated AP are important, see Figures 7C,G. When looking at the resting membrane potential (RMP) and the APD at 90% repolarisation (APD_90_) for example, RMP is increased from −88 mV to −82 mV for the TTP06 model, and from −88 mV to −83 mV in the ORd-CiPA model when increasing Γ_0_ by 30 mM. APD_90_ is increased from 299 to 306 ms for the TTP06 model, and is increased from 265 to 273 ms in the TTP06 ORd-CiPA model, when increasing Γ_0_ by 30 mM.

Figures 7D,H show that Γ_0_ has an effect on the APD_90_ steady state restitution portraits. The bifurcation of APD_90_ in the restitution portrait is particularly important as it is characteristic of *alternans*, when two consecutive APs do not have the same APD_90_ but the model outputs are still periodic. Note that when stable alternans occurs, the limit cycle no longer follows the trajectory of the state variables over a single pacing period, but over two consecutive pacing periods.

There is a bifurcation of APD_90_ for pacing periods at 700 ms for the TTP06 model and at 400 ms for the ORd-CiPA model. The pacing periods generating this bifurcation appear to be independent of Γ_0_. However, the steepness of the restitution slope as well as the size of the bifurcation depend on Γ_0_ used for the simulation, especially for the ORd-CiPA model. In the studied models, higher values of Γ_0_ generate wider bifurcations in the APD_90_ restitution portrait. The impact of Γ_0_ on characteristics of the alternans predicted by the TTP06 and ORd-CiPA models stresses the need to carefully consider the value of Γ_0_ used in AP models.

## 4 Calibration of AP Models and Γ_0_

The dependency of model outputs to Γ_0_ observed in Figure 7 is also expected have an impact when fitting parameter values to whole traces of *V*_m_, or their derived biomarkers. Indeed, if Γ_0_ is fixed to a value that incorrectly summarises the experimental concentrations under which the data were generated, we might expect a fitting process to return parameter values which are skewed away from their correct values. A fitting of the ORd-CiPA model to synthetic (simulated) data was performed to examine this effect.

The synthetic datasets used in model training were generated by running the ORd-CiPA model for 2000 pre-paces (1 Hz pacing), and recording the 2001th AP, with one data point per 0.05 ms, no noise was added. The “true” scaling parameters for conductances were then “forgotten” and re-calibrated to the synthetic AP data, as in Johnstone et al. (2016). The parameters used for the simulations are expressed as: *g*_simulation_ = *θ* × *g*_original_, with g_simulation_ the value of the conductance used for the simulation, *θ* the scaling factor, and g_original_ the original value of the conductance parameter. Thus, a scaling factor of *θ* = 1 corresponds to the conductance used in the original published model (the “*true*” value in this synthetic study).

Three cases were explored to assess the influence of Γ_0_ in the fitting process. In the first case, the initial conditions were unaltered (assumed to be known/exactly correct), therefore the value of Γ_0_ during the fitting was set to the “true” value, i.e. the one used for synthetic data generation. In the second case, the model was fitted with a fixed and incorrect Γ_0_ value computed from initial concentrations and voltage published for the TTP06 model, a different but still plausible value. The third fitting is the same as the second case, but Γ_0_ was added to the set of parameters to be fitted, to allow compensation for discrepancy in the initial intracellular ion concentrations provided by the user (in terms of Figure 6 this allows flexibility in the plane upon which intracellular concentrations will settle). The initial conditions used for the fittings are reported in the Table 2.

**TABLE 2:** Initial conditions used in the various fittings of the ORd-CiPA model to synthetic data.

Case	Γ_0_	Initial conditions for ${\left[K^{+}\right]}_{i}$	Initial conditions for other concentrations
Data generation	− 7.801	144.6 mM	ORd-CiPA
#1 Fixed & ‘correct’ Γ_0_	− 7.801	144.6 mM	ORd-CiPA
#2 Fixed & ‘wrong’ Γ_0_	− 1.562	135.4 mM	TTP06
#3 Fitted Γ_0_	Fitted	135.4 mM	TTP06

When using initial concentrations from the TTP06 model, calcium concentrations, ${\left[{\text{Na}}^{+}\right]}_{\text{i}}$ and ${\left[K^{+}\right]}_{i}$ were set to the values published by Ten Tusscher and Panfilov (2006) [K^+^]_SS_ and [Na^+^]_SS_ were initialised to the same value as ${\left[K^{+}\right]}_{i}$ and ${\left[{\text{Na}}^{+}\right]}_{\text{i}}$ . In the ORd-CiPA model, the SR is split into two sub-compartments while the TTP06 model has only one SR compartment. Therefore [Ca^2+^]_NSR_ and [Ca^2+^]_JSR_ were initialised at the same concentration published by Ten Tusscher et al. for [Ca^2+^]_SR_.

The optimisation problem was defined as the minimisation of the sum of square errors between the synthetic data and the fitted model AP. The fitting algorithm uses the PINTS Python package (https://github.com/pints-team/pints) (Clerx et al., 2019), to run the Covariance Matrix Adaptation-Evolution Strategy (CMA-ES) (Hansen et al., 2003). The scaling factor parameters *θ*_CaL_, *θ*_Kr_, *θ*_Ks_, *θ*_Na_, *θ*_NaL_ of the ORd-CiPA model were fitted. The initial guesses for scaling factors were taken from the range 0.2–5, while the boundaries were set to 0.1 to 10. The CMA-ES hyper-parameter Σ_0_, the initial proposal covariance for new parameter samples, was set to 0.1 along the diagonal for all parameters and zero otherwise.

**FIGURE 8:**
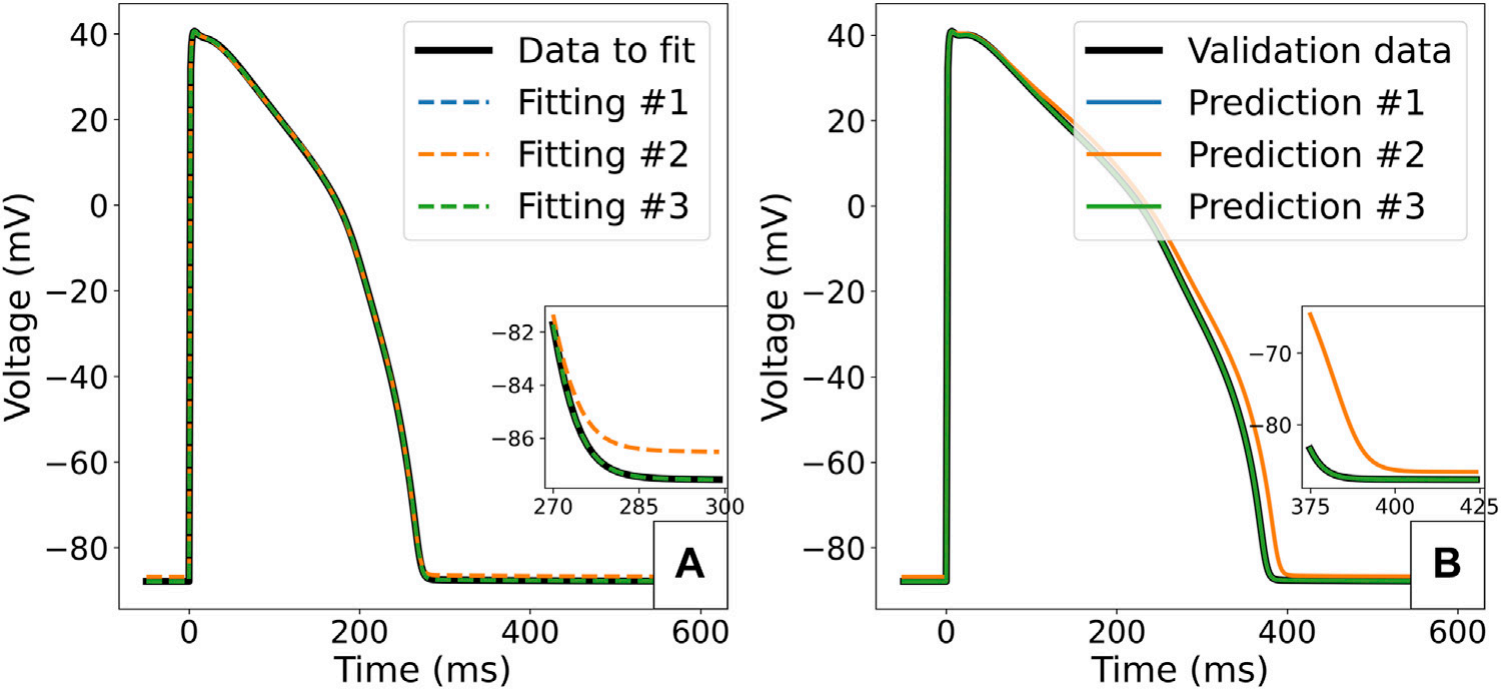
Predicted APs for the ORd-CiPA model fitted to synthetic data. **(A)** Comparison of the synthetic data with APs obtained from optimal parameterisations in the different fitting cases. **(B)** Prediction of response of the model to 50% block of *I*_*Kr*_. Predictions of model with parameter fittings #1, #3 and the true parameters set overlay.

The value of scaling parameters retrieved by the three fittings are compared in Table 3, and the corresponding APs are plotted in Figure 8. In the case of the first fitting with the correct Γ_0_, the true parameter values are retrieved as expected due to these model parameters being identifiable. In the case of the second fitting with a discrepancy in Γ_0_, the model cannot converge to the right limit cycle. The optimal AP is still very similar to the synthetic data, the only difference being a small shift in the resting membrane potential, as seen in Figure 8A. However, the discrepancy in ionic concentrations is compensated by a dramatic shift in the retrieved scaling parameters, especially for *g*_Ks_ (0.522) and *g*_NaL_ (1.585). This impacts the response of the model to perturbation: for example 50% block of *I*_Kr_ as shown in Figure 8B, where we see a 14 ms difference in the predicted APD_90_ which would be significant in many drug effect prediction settings.

**TABLE 3:** Parameters retrieved from fittings in the investigated cases. The fitting process with an incorrect Γ_0_ value yields incorrect values for model parameters. Such a model suffers from poor predictive power, this can be corrected by fitting Γ_0_ together with the other model parameters.

Case	Γ_0_ (mM)	Diastolic [K+]_i_ atlimitcycle	θ_CaL_	θ_Kr_	θ_Ks_	θ_Na_	θ_NaL_	APD_90_ baseline	APD_90_ with 50% I_Kr_ block
**Data generation**	−**7.801**	**144.4**	**1**	**1**	**1**	**1**	**1**	**266** ms	**369** ms
#1 Fixed & “correct” Γ_0_	−7.801	144.4	1.000	1.000	1.000	1.000	1.000	266 ms	369 ms
#2 Fixed & “wrong” Γ_0_	−1.562	138.6	0.760	1.187	0.522	1.129	1.585	265 ms	383 ms
#3 Fitted Γ_0_	−7.801	144.4	1.000	1.000	1.000	1.000	1.000	266 ms	369 ms

Values associated with the synthetic (simulated) data generation are written in bold font.

In the case of the third fitting with Γ_0_ as an inferred parameter, the true values for all scaling parameters could be recovered. The fact that the value of Γ_0_ could also be accurately retrieved from fitting supports its identifiability as a model parameter, at least in the absence of model misspecification/discrepancy.

### 4.1 Calibration When Multiple Stable Limit Cycles Exist for a Single Γ_0_ Value

It was shown in Section 3.3 that the ORd-CiPA model, with published parameters, has a unique limit cycle for any particular value of Γ_0_ that has been used (implicitly) in previous models. As shown by previous studies, under certain conditions there are possibly multiple quiescent steady state (Guan et al., 1997; Jacquemet, 2007) and/or limit cycle (Surovyatkina et al., 2010) solutions for the same value of Γ_0_.

**FIGURE 9:**
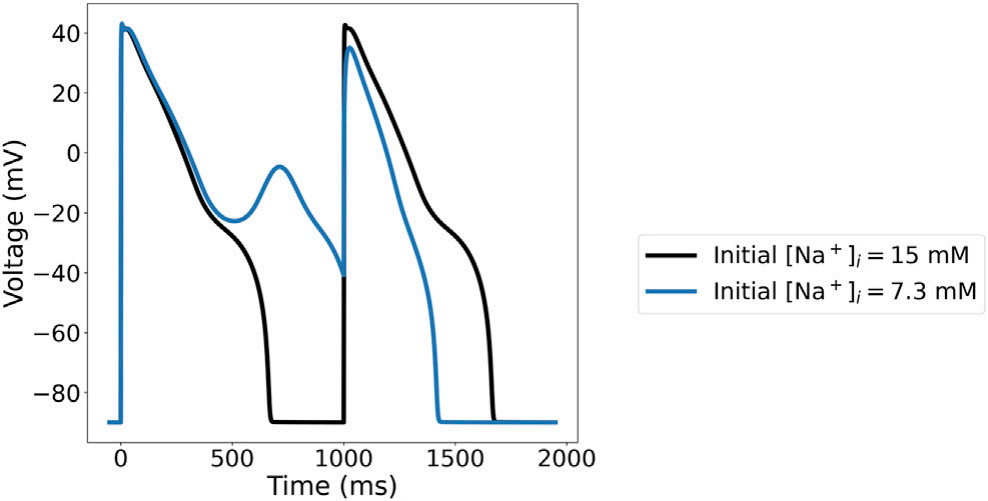
Limit cycle APs for the ORd-CiPA model under 95% of I_Kr_ reduction, generated with the same value for Γ_0_=−20 mM, but different initial Na^+^ concentrations. With the initial Na^+^ concentration set to 15 mM (Black), the limit cycle AP shows no early after-depolarisation (EAD). With a lower initial Na^+^ concentration of 7.3 mM (Blue), the limit cycle AP exhibits alternans with an EAD.

For instance, with 95% reduction of I_Kr_, Γ_0_ = −20 mM, and 1 Hz pacing, the ORd-CiPA model has two stable limit cycle APs, shown in Figure 9. With the initial Na^+^ concentration as originally published in the ORd-CiPA model, the limit cycle AP has an early after-depolarisation (EAD), whereas the limit cycle AP with higher initial Na^+^ concentration exhibits alternans and an EAD. This is characteristic of a bifurcation of the limit cycle for the same value of Γ_0_, which is investigated further in this section.

Various conditions of I_Kr_ block (0, 90 and 95%) were applied to the ORd-CiPA model to test for the presence of multiple limit cycles for a single value of Γ_0_. As in Section 3.3, the ORd-CiPA model was paced to its limit cycle for various initial conditions that sample the physiological range of concentrations reported in Section 3.2, but variations of initial conditions were considered only for ${\left[K^{+}\right]}_{i}$ and ${\left[{\text{Na}}^{+}\right]}_{\text{i}}$ this time. Given the low influence of [Ca^2+^] variations on Γ_0_ value, its influence on the model outputs were neglected. Eq. 6 defines a linear relationship between ${\left[{\text{Na}}^{+}\right]}_{\text{i}}$ and ${\left[K^{+}\right]}_{i}$ and Γ_0_, and therefore for a fixed value of Γ_0_, the intracellular concentrations follow a line in the ( ${\left[{\text{Na}}^{+}\right]}_{\text{i}}$ , ${\left[K^{+}\right]}_{i}$ ) plane, if the other ionic concentrations are not changed. Ten different initial conditions were sampled for each of the 15 values of Γ_0_ covering the physiological range of concentrations ( ${\left[K^{+}\right]}_{i}$ between 120 and 152 mM and ${\left[{\text{Na}}^{+}\right]}_{\text{i}}$ between 4 and 16 mM). In case there is alternans, diastolic concentrations are read out at the end of the longer AP.

**FIGURE 10:**
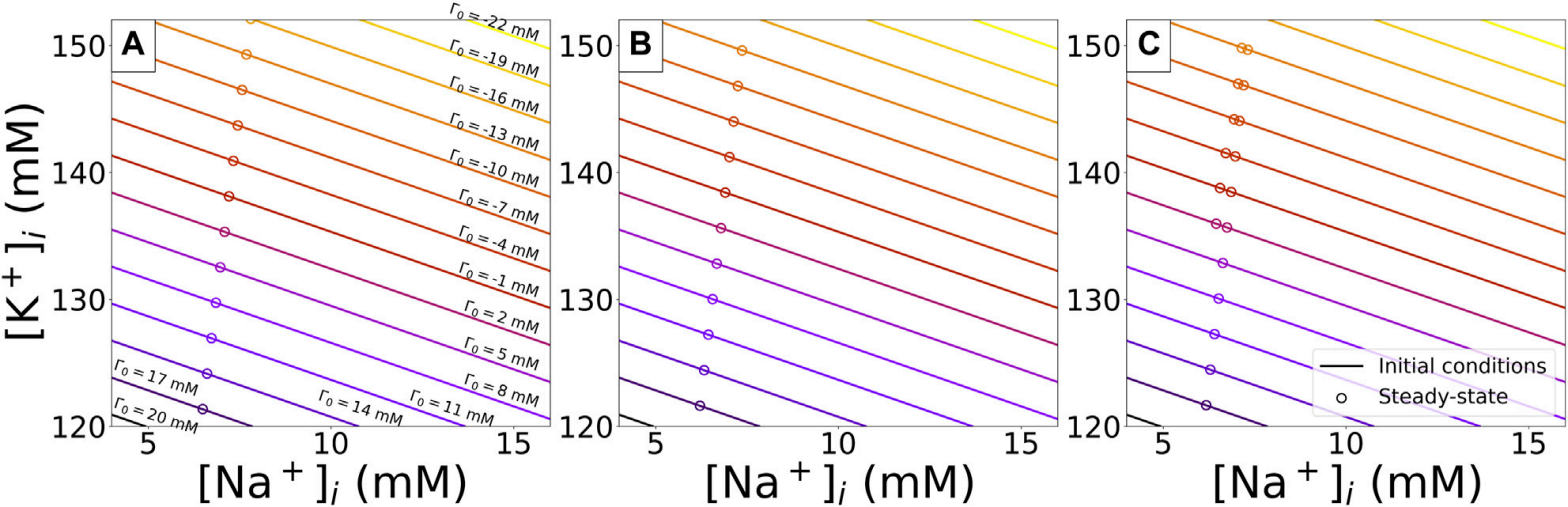
Limit cycle concentrations of ${\left[K^{+}\right]}_{i}$ and ${\left[{\text{Na}}^{+}\right]}_{i}$ for simulations with ORd-CiPA model starting from different initial conditions. Each line corresponds to combinations of intracellular concentrations bound by a single Γ_0_ value. For each value of Γ_0_, 10 combinations of ${\left[K^{+}\right]}_{i}$ and ${\left[{\text{Na}}^{+}\right]}_{i}$ are used to sample the whole physiological range reported in Section 3.2. Limit cycle concentrations of the 10 combinations are marked by circles, with colour matching the initial conditions. For *I*_*Kr*_ reduction up to 90%, a unique limit cycle can be reached per value of Γ_0_. In the case of 95% of *I*_*Kr*_ reduction, two distinct limit cycles can be observed for higher intracellular concentrations. **(A)**: With no *I*_*Kr*_ reduction. **(B)**: With 90% *I*_*Kr*_ reduction. **(C)**: With 95% *I*_*Kr*_ reduction.

The limit cycle diastolic concentrations reached for the various Γ_0_ values with various *I*_Kr_ block conditions are represented in Figure 10. For *I*_Kr_ block lower than 90% across the range of initial conditions we studied, the limit cycle is unique for a given value of Γ_0_. In such situations, fitting Γ_0_ would be sufficient to fully inform the intracellular concentrations.

In the extreme case of 95% of I_Kr_ block, a bifurcation is observed for the ORd-CiPA model—see Figure 10C. A second stable limit cycle appears, and intracellular concentrations converge to one or the other limit cycle value depending on their initial conditions, despite corresponding to the same Γ_0_ value. The multiple limit cycles at a fixed Γ_0_ value are observed for Γ_0_ values ranging from −13 to 2 mM—see Figure 10C. In such cases, Γ_0_ does not solely determine which limit cycle will be reached, and one needs to consider ${\left[K^{+}\right]}_{i}$ and ${\left[{\text{Na}}^{+}\right]}_{\text{i}}$ initial conditions.

As observed in Figure 10, multiple stable limit cycles can be found for the same value of Γ_0_ under particular conditions. In this section, we investigate how the bifurcations of the limit cycle can impact the fitting process. Under 95% of I_Kr_ reduction, there are two stable limit cycle APs for the ORd-CiPA model for the same value of Γ_0_: one with early after-depolarisation (EAD) generated with low initial ${\left[{\text{Na}}^{+}\right]}_{\text{i}}$ , and one without EAD when simulating the limit cycle AP from high initial ${\left[{\text{Na}}^{+}\right]}_{\text{i}}$ —see Figure 9.

The synthetic data was generated with the ORd-CiPA model under 95% of I_Kr_ block, with intracellular concentrations initialised at ${\left[{\text{Na}}^{+}\right]}_{\text{i}}\;=15$ mM and ${\left[K^{+}\right]}_{i}$ = 149 mM, corresponding to Γ_0_ = −20 mM. Synthetic data showed no EAD. As seen in Figure 9, there is a second stable limit cycle AP, with EAD, in this configuration of the ORd-CiPA model with lower initial Na^+^ concentration.

During the fitting process, the initial concentration of Na^+^ was fixed to its published value ${\left[{\text{Na}}^{+}\right]}_{\text{i}}\;=7.3$ mM, and when a new value of Γ_0_ was proposed by the fitting algorithm, the changes in Γ_0_ were attributed to K^+^ ions. As a consequence, when the “true parameters” were evaluated during the fitting process, an EAD was observed. The fitting of the ORd-CiPA model to synthetic data from the same model was performed with the same methods as in Section 4. The same parameters as previously were fitted (*θ*_CaL_, *θ*_Kr_, *θ*_Ks_, *θ*_Na_, *θ*_NaL_, Γ_0_).

Note that for Γ_0_ = −20 mM, ${\left[{\text{Na}}^{+}\right]}_{\text{i}}$ can take only values between 14 and 16 mM for ${\left[K^{+}\right]}_{i}$ to remain in the physiological range (Figure 10). This bifurcation was selected despite the initial and limit cycle concentrations being outside the physiological range, because of the dramatic changes between the two limit cycle APs that make more visual the potential impact of multiple stable limit cycles on the parameters retrieved from model calibration.

**FIGURE 11:**
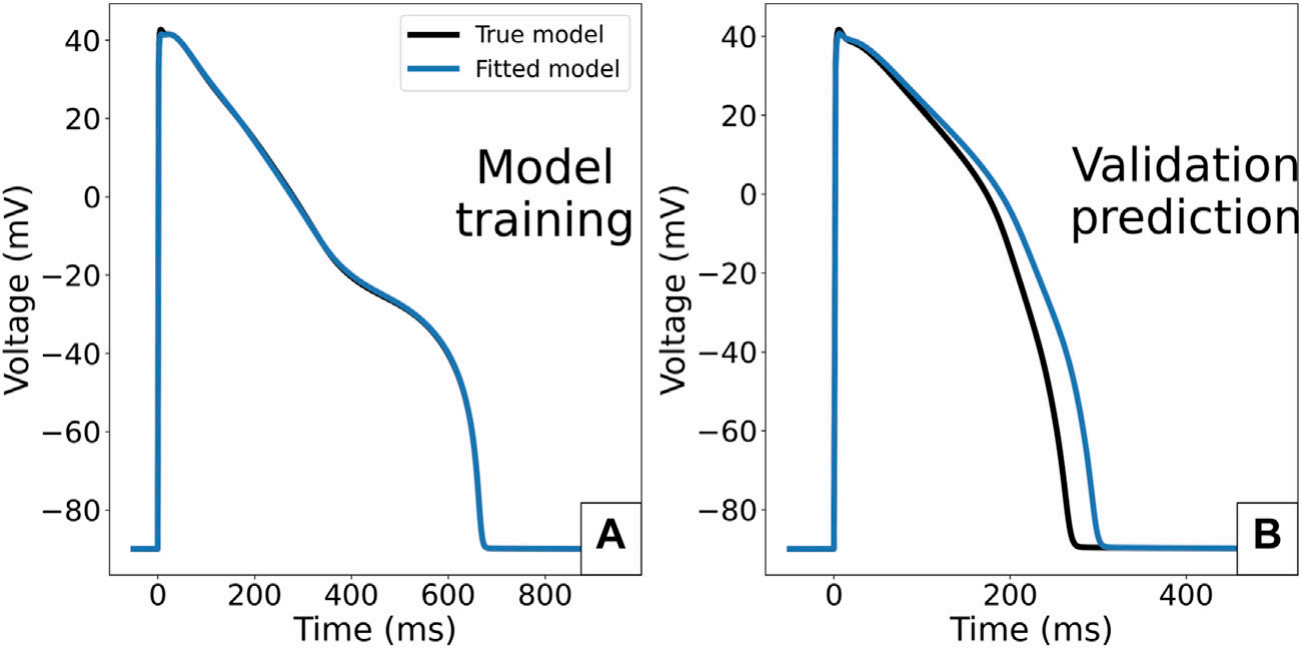
Consequence of fitting the ORd-CiPA model in case of multiple stable limit cycles for the same Γ_0_ value. **(A)**: ORd-CiPA fitted with initial ${\left[{\text{Na}}^{+}\right]}_{i}=7.3$ mM under 95% of *I*_*Kr*_ block is able to reproduce the synthetic data generated with initial ${\left[{\text{Na}}^{+}\right]}_{i}=15$ mM (APs superposed). **(B)**: predictions for no I_Kr_ block. Despite the good fit to I_Kr_ block data in panel **(A)**, incorrect parameter values are retrieved from fitting, and the prediction of the calibrated model is erroneous.

The parameters retrieved from the fitting are reported in Table 4. The limit cycle AP under 95% I_Kr_ reduction for the calibrated model is compared to the synthetic data (Figure 11A) and its prediction of AP without I_Kr_ block is compared to that of the true model that generated the synthetic data in the validation case of Figure 11B.

**TABLE 4:** Rescaling factors for conductance parameters retrieved from fitting to data generated under conditions where several stable limit cycles coexist for the same value of Γ_0_ = −20 mM.

	Γ_0_ (mM)	Diastolic [K+]_i_ (mM)	θ_CaL_	θ_Kr_	θ_Ks_	θ_Na_	θ_NaL_	APD_90_ with 95% I_Kr_ block	APD_90_ baseline
**Data generation**	**−20.0**	**156.18**	**1**	**1**	**1**	**1**	**1**	**663 ms**	**264 ms**
Fitted values	**−**19.7	155.73	0.863	0.933	1.263	0.936	1.574	663 ms	294 ms

Values associated with the synthetic (simulated) data generation are written in bold font.

The optimal values of *θ*_Kr_ and *θ*_Na_ are close to their true values, but *θ*_NaL_ and *θ*_Ks_ have considerable differences to their true values, 57 and 26% too large respectively. This explains why even though the synthetic data AP is well reproduced (Figure 11A), the fitted model makes an incorrect prediction in the validation case with no *I*_Kr_ block (Figure 11B). The optimal value of Γ_0_ is interestingly close to its true value. However, one cannot conclude from this example alone that Γ_0_ value will still be correctly recovered in the case of bifurcation.

In this case with bifurcation, fitting initial conditions for both ${\left[{\text{Na}}^{+}\right]}_{\text{i}}$ and ${\left[K^{+}\right]}_{i}$ would be necessary to reach the correct limit cycle and obtain a correct optimal model. However, we would not recommend fitting both ${\left[{\text{Na}}^{+}\right]}_{\text{i}}$ and ${\left[K^{+}\right]}_{i}$ simultaneously as a standard. In most cases, there is only one limit cycle solution for a given value of Γ_0_, so that the two parameters would be unidentifiable (see Whittaker et al., 2020).

## 5 Discussion

We investigated the consequences of computing voltage in AP models directly from concentrations, using an algebraic-*V*_m_ formulation (Eq. 6). This method for computing voltage increases the numerical accuracy of solutions, compared to the canonical derivative-*V*_m_ method of integrating the sum of trans-membrane currents. The computation time of simulations is not impacted significantly by the choice of expression for the voltage. Changing to the algebraic-*V*_m_ form of the model did not reduce the computational time required for AP simulations, as it does not change the stiffness of the model (the main driver for the computational cost).

Γ_0_ is a constant representing the net concentration of un-modelled charge present in the model, needed to ensure the consistency of initial values for concentrations and voltage. In most cases, the value of Γ_0_ defines the steady-state behaviour of the model, regardless of the combination of initial values for state variables such as concentrations in the simulations. Given the high variability of intracellular concentrations that have been used in action potential models, with less variability in extracellular concentrations, Γ_0_ is also highly variable. Extreme variations of Γ_0_ lead to very different steady-state behaviours and substantially impact their outputs, making it important to establish the value of Γ_0_ as accurately as possible.

Measurements of intracellular ionic concentrations in intact myocytes are not generally available alongside recordings of electrophysiological activity used to calibrate AP models. We showed that this issue could potentially be addressed by inferring Γ_0_ from the data, along with other parameters of the AP model.

With the algebraic-*V*_m_ form of the model, the algebraic constraint on the variables appears explicitly. At each time-step, this constraint is therefore rigorously applied to the system. With the derivative-*V*_m_ form of the model, the constraint is mathematically satisfied by the system—by design in AP models which satisfy the conservation of charge principle—but during the numerical integration of the equations, the constraint is not verified at each time step. Therefore, the numerical errors that appear during the integration allow the constraint to be violated. This violation of conservation of charge explains that with a coarse solver tolerance, the model does not properly converge to a limit cycle—see Figure 2. Livshitz & Rudy noted that AP models are often mistaken as Ordinary Differential Equation (ODE) systems when they are actually Differential-Algebraic Equation (DAE) systems—ODE systems with algebraic constraints. With the algebraic-*V*_m_ form of the model, all constraints of the DAEs appear explicitly, which is best practice (Livshitz and Rudy, 2009). In theory, the differential and algebraic representations of the membrane voltage are still mathematically equivalent, so modellers could use either of them as preferred (Hund et al., 2001). In practice, we recommend to use the algebraic-*V*_m_ formulation.

Using the algebraic-*V*_m_ form of the model makes also Γ_0_ appear as a model parameter, highlighting the need to consider its value explicitly. We propose to infer Γ_0_ from the experimental data on which the model is calibrated. Endresen et al. (2000) reported with the derivative-*V*_m_ form of the model that “the observer tracks only the variations in the number of ions, but then an initial concentration must be guessed”. Livshitz & Rudy proposed criteria for validation against experimental data and adequate comparison between dynamic models (Livshitz and Rudy, 2009). Among these criteria, the use of “a consistent set of initial conditions for state variables (*V*_m_, intracellular ion concentrations)” is recommended. Smirnov et al. (2020) also noted that the question of initial conditions for ionic concentrations is often overlooked when fitting AP models, when they fitted the O’Hara Rudy model (O’Hara et al., 2011) to AP recordings from optical mapping experiments in human ventricular wedges.

The errors induced in conductance fits when using a fixed but incorrect Γ_0_—see Section 4—emphasise the importance of using the correct initial conditions for concentrations when fitting to AP data. An AP model calibrated using an incorrect representation of concentrations (i.e. an incorrect but plausible value for Γ_0_) is badly parameterised with up to ±50*%* error in some maximal conductance parameters, and has a reduced predictive power.

Our results show that Γ_0_ can be fitted to compensate for errors in assumed intracellular concentrations, at least when fitting to synthetic (simulated) AP data. So we recommend inferring Γ_0_ from the training data during model calibration, following the methods of Section 4. When using real data, discrepancy in the AP model may cause additional problems, but still the possibility for uncertainty in Γ_0_ should be explicitly considered.

In our study, we show that due to the conservation law: 1) a consistent Γ_0_ value should be used throughout the model calibration, and 2) it is sufficient to fit the value of Γ_0_ to capture the input of intracellular concentrations on steady state outputs, unless bifurcations are present. The second point is supported by observations on other models reported in the literature (Hund et al., 2001; Jacquemet, 2007; Livshitz and Rudy, 2009; Pan et al., 2018). For example, Smirnov et al. (2020) have included initial values for ${\left[{\text{Na}}^{+}\right]}_{\text{i}}$ and ${\left[{\text{Ca}}^{2+}\right]}_{\text{SR}}$ in their set of parameters to calibrate, which is similar to fitting Γ_0_. However, they fitted their initial conditions independently at each pacing rate, thus changing the value of Γ_0_ from one pacing rate to another.

It remains important to consider that the uniqueness of the limit cycle for a single Γ_0_ value cannot be always guaranteed (Guan et al., 1997; Jacquemet, 2007). The methods presented in Section 3.3 can be reused to verify that Γ_0_ solely defines the limit cycle for a model under a given set of studied experimental conditions. If the uniqueness of a limit cycle is verified, it is reasonable to fit Γ_0_ alone to summarise the initial conditions of intracellular ionic concentrations. Otherwise, in case of bifurcation of the limit cycle, we would recommend fitting Γ_0_ and the initial condition of ${\left[{\text{Na}}^{+}\right]}_{\text{i}}$ . Alternatively, initial conditions for two intracellular concentrations could be inferred, for instance ${\left[K^{+}\right]}_{i}$ and ${\left[{\text{Na}}^{+}\right]}_{\text{i}}$ which have the highest contribution to the value of Γ_0_.

### 5.1 Limitations

As mentioned above and in the literature (Guan et al., 1997; Jacquemet, 2007), the uniqueness of the steady states for a single Γ_0_ value is not always guaranteed. In cases of bifurcation, where several stable solutions exist for the model with a single value of Γ_0_, Γ_0_ (as well as other parameters) can be incorrectly determined. We observed in this study that for the ORd-CiPA model, the limit cycle is unique in most physiologically-plausible cases. However, this property does not always hold if parameters are changed. A method to investigate thoroughly the uniqueness of the limit cycle for a given value of Γ_0_ for all parameterisations of an AP model could be extremely costly computationally. Still, we have demonstrated for the ORd-CiPA model, as originally published, that Γ_0_ is identifiable and could be correctly estimated. We observed consistent findings for Γ_0_ in the TTP06 model, which has a very different model structure to the ORd-CiPA model—data not shown. We therefore expect this behaviour to be replicated for all AP models that conserve charge. Hence we recommend to consider calibrating Γ_0_ as a parameter that usually encapsulates both the initial conditions of the modelled ionic species and the un-modelled charge. In the cases where there are multiple steady states for the same Γ_0_, the unidentifiability could be resolved by fitting initial conditions for ionic concentrations as well.

To define the physiologically-plausible range of concentrations, we used the extreme values of ${\left[K^{+}\right]}_{i}$ reported in previous human ventricular AP models. Direct experimental measurements of ${\left[K^{+}\right]}_{i}$ would help refining this range. Moreover, ${\left[{\text{Na}}^{+}\right]}_{\text{i}}$ and ${\left[K^{+}\right]}_{i}$ were considered separately in our study. Simultaneous experimental measurements of ${\left[{\text{Na}}^{+}\right]}_{\text{i}}$ and ${\left[K^{+}\right]}_{i}$ in human ventricular cardiomyocytes would give better understanding of correlation between these concentrations, which may further restrict the range of physiologically-plausible Γ_0_ values.

When AP models are used to investigate changes in extracellular concentrations (e.g. when simulating hypo/er-kalemia or ischaemia—pathological changes to extracellular concentrations such as [K^+^]) care is needed with Eq. 6. In such situations, as the extracellular ion of interest changes concentration, opposite charges will be introduced into the same solution to maintain electrical neutrality (e.g. if we experimentally use the salt KCl to change ${\left[K^{+}\right]}_{o}$ we also change ${\left[{Cl}^{-}\right]}_{o}$ ); if one ion is accounted for in Eq. 6 but the ‘opposite ion’ is not (e.g. the model does include ${\left[K^{+}\right]}_{o}$ but does not explicitly consider ${\left[{Cl}^{-}\right]}_{o}$ ) then Γ_0_ will need to be adjusted by the same amount to account for this extra “opposite” charge. For models where external concentrations are fixed as constants, an equation of the form of Eq. 2 with *V*_0_ or *C*_0_ can then be used equivalently, and would simplify simulation procedures when extracellular concentrations are changed by the user, but the interpretation of Γ_0_ as “net un-modelled charge” is clearer.

### 5.2 Possible Extensions to This Study

Although this study was focused on ventricular AP models, the conservation law that binds together the voltage and intracellular ionic concentrations applies to all cellular electrophysiology models: other cardiac cell types, neural, gastric, skeletal muscle etc.

The improvement in numerical accuracy enabled by the algebraic-*V*_m_ form of the model was shown to reduce the numerical error that can lead to deviation of state variables after reaching the periodic steady state—see Figure 3 and Supplementary Material Section S1.4. The computational efficiency was similar with the algebraic-*V*_m_ form of the model when using the same solver tolerance.

The extent to which intracellular concentrations are well established has been somewhat overlooked (Smirnov et al., 2020). Our study, showed the importance of the correct estimation of Γ_0_ in specifying concentrations. In literature models, there is significant variation between the assumed initial concentrations, and therefore variation in Γ_0_, as shown in Section 3.2. In papers on action potential model development, we have not found any discussion of the choice of Γ_0_, or equivalently the choice of the offset between concentrations and voltage in initial conditions, perhaps suggesting somewhat arbitrary choices. It remains to be seen whether Γ_0_ exhibits significant physiological variation to contribute to inter-cell and/or inter-individual differences in electrophysiology, or whether it is a well-constrained biological quantity—which would be the case if the un-modelled missing ions that Γ_0_ represents do not vary significantly between cells or individuals. In either case, Γ_0_ strongly influences model behaviour and a concerted effort should be made to identify its value alongside other key model parameters. The recent emergence of cell-specific models (Groenendaal et al., 2015) may offer an approach to quantify Γ_0_ more accurately.

## 6 Conclusion

We advocate here for the use of the algebraic-voltage form of AP models, as it improves the stability of numerical solutions by enforcing a hidden algebraic constraint in the models. Furthermore, the algebraic-voltage form ensures that the model conserves charge. It also requires the modeller to think carefully about initial conditions for intracellular concentrations and to acknowledge their effects on the model output. We recommend consideration of the potential discrepancy and uncertainty in intra- and extracellular concentrations of ions, as model outputs and model fitting are dependent on these. The Γ_0_ value summarises these factors into one parameter which can be fitted alongside the rest of a model.

## Data Availability

The datasets presented in this study can be found in online repositories. The names of the repository/repositories can be found below: Github: https://github.com/CardiacModelling/Gamma_0. Zenodo (permanent archive of Github): http://dx.doi.org/10.5281/zenodo.6423387.
