# Supplementary material for: A Parameter Representing Missing Charge Should Be Considered when Calibrating Action Potential Models
Source: Front Physiol. 2022 Apr 26;13:879035. doi: 10.3389/fphys.2022.879035 (PMC9086858; doi:10.3389/fphys.2022.879035)
Supplement: Supplementary file 1 [file Presentation1.pdf]

frontiers

Supplementary Material for: “A parameter representing missing charge should be considered when calibrating action potential models”

S1 SUPPLEMENTARY MATERIAL

S1.1 Units correction in Ten Tusscher-Panfilov 2006 (TTP06) CellML File

Inconsistencies in units were observed between the TTP06 CellML file taken from the CellML repository and the Ten Tusscher and Panfilov (2006) paper where the model was described. However, the CellML model is consistent with the original C code published by Ten Tusscher on her website. The units and values corrected for the present study are summarised in Table S1 below.

Table S1. Correction of the units of Ten Tusscher-Panfilov 2006 model from the CellML repository

Parameter	Published model value	Corrected value
F	96485 $C.mmol^{-1}$	96.485 $C.mmol^{-1}$
R	8314.472 $J.mol^{-1}.K^{-1}$	8.314472 $J.mol^{-1}.K^{-1}$
C_m	0.185 μF	1.85 $\times 10^{-4} \mu F$
V_c	0.016404 μm^3	0.016404 nL
V_{sr}	1.094 $\times 10^{-3} \mu m^3$	1.094 $\times 10^{-3} nL$
V_{ss}	5.468 $\times 10^{-5} \mu m^3$	5.468 $\times 10^{-5} nL$
G_{CaL}	3.98 $\times 10^{-5} L.F^{-1}.s^{-1}$	3.98 $\times 10^{-2} L.F^{-1}.s^{-1}$

In most of the equations involving F or R, these parameters are used in the ratio $\frac{RT}{F}$, so the rescale of both by a factor 1/1000 did not change anything in the model solving. The only exception is the computation of I_{CaL} , the L-type calcium current, where the ratio $\frac{RT}{F^2}$ is used. This is compensated by the rescale of the conductance G_{CaL} by a factor 1000.

The total membrane capacitance of TTP06 was modelled initially with the value $C_m = 0.185 \mu F$ which is orders of magnitude away from the experimental $\mathcal{O}(100pF)$, consistent with the corrected value.

The volumes of the cell compartments were not physiological in μm^3 and match much better with experimental knowledge when corrected to nL.

These changes have been made to the CellML file in the Physiome Model Repository (Yu et al., 2011).

S1.2 Comparison of algebraic voltage equations

Studies from the literature proposed integration constants in their algebraic expression of V_m that take into account the charge of omitted species in various ways. The integration constant binding the ionic concentrations and the membrane voltage took various forms in the literature and represented slightly different concepts: V_0 (Varghese and Sell, 1997; Endresen et al., 2000), C_0 (Hund et al., 2001), Q_{ns} (Jacquemet, 2007; Livshitz and Rudy, 2009). These constants have essentially the same modelling properties, as they involve writing the model using the algebraic voltage expression to satisfy a conservation law. The integration constants in the algebraic voltage equations reported here are compared in Table S2 in the Supplementary Materials.

The transmembrane voltage is generated by a difference of electrical potential between the intra- and extracellular spaces. As such, the integration constants in the algebraic expression of V_{m} account for all the electrically-charged species, and their expression and value depend on the species that are included in the model. The complexity of the interpretation of these constants is therefore due to the fact that the extracellular space does not have a null electrical charge, contrary to Jacquemet's assumption when computing Q_{ns} (Jacquemet, 2007).

To compute the "non-specific charge Q_{ns}" as defined by Jacquemet (2007) (same as Q_0 from Livshitz and Rudy (2009)), the external concentrations are omitted:

$$C_{\mathrm{m}}V_{\mathrm{m}} = \mathcal{V}_{\mathrm{i}}F\sum_X\sum_k\frac{\mathcal{V}_k}{\mathcal{V}_{\mathrm{i}}}z_X[X]_{\text{tot, k}} + Q_{ns}. \tag{S1}$$

Varghese & Sell, as well as Jacquemet (Jacquemet, 2007), noted that the "principle of Faraday: Q = CV, rewritten in terms of ionic concentrations rather than the charge" applies (Varghese and Sell, 1997). Eq. S1 is the direct application of the Faraday equation to models where extracellular concentrations are constant.

$C_{\mathrm{m}}V_{\mathrm{m}}$ corresponds physiologically to the difference in total charge across the cell membrane or the *net charge* of the cell. The double sum of Eq. S1 corresponds to the total charge of modelled species across all intracellular compartments, and Q_{ns} is therefore the sum of the remaining charges: non-modelled intracellular charges and total extracellular charges.

Similarly to Q_{ns}, the constant C_0, as defined by Hund et al. (2001), includes the total concentration non-modelled charges and the concentration of extracellular charges for modelled species. C_0 can be understood as the concentration of charge leading to Q_{ns}, so it suffers from the same limited physiological meaning.

Table S2 compares the various expression used in the literature to express the integration constant arising from the conservation principle. Note that in the expressions by Varghese and Sell (1997), the constant is labeled C_0 but is consistent with a voltage. Also, in their expressions, the volumes of the compartments of the junctional and network sarcoplasmic reticula were omitted. We corrected these omissions in the present piece of work, in particular when writing Eq. 2 (main text).

Table S2. Table comparing the different expressions of the integration constant in the algebraic voltage equations from literature.

Reference	Label	Expression	Note
Varghese and Sell (1997)	C_0	No expression	Did not explore it further, focused on consequences of the implicit conservation law.
Endresen et al. (2000)	V_0	$v_0 = -\frac{F\mathcal{V}_\text{i}}{C_\text{m}}([\text{K}^+]_\text{ext} + [\text{Na}^+]_\text{ext} + 2[\text{Ca}^{2+}]_\text{ext})$	v_0 expressed as voltage offset.
Hund et al. (2001)	C_0	No expression	C_0 computed so that initial conditions for intracellular concentrations match with initial voltage in the model.
Jacquemet (2007)	Q_{ns}	$Q_{ns} = \mathcal{V}_\text{i}F[NS]_\text{i}$	$[NS]_\text{i}$ corresponds to the non-specific charge concentration. Can be computed following Endresen or Hund approach.
Livshitz and Rudy (2009)	Q_0	$C_\text{m}V = -Q_0 + Q_\text{stim} + F\sum_X \sum_y \mathcal{V}_k z_X [X]_y$	"Q_0 accounts for charge contributed by nonspecific, mainly anionic, charged intracellular molecules (e.g. impermeable proteins)".

S1.3 Changing the voltage expression of a model to the algebraic expression

Changing to the algebraic expression for voltage in the model requires to account exactly for all the charged species included in the model. This ensures that the model satisfies the conservation of charge principle, but requires extra caution when building and using AP models. Therefore, we aim at providing in this section the reader with help on how to proceed.

S1.3.1 Computation of total Ca^{2+} concentration

To compute Γ_0 in a model, it is necessary to compute the total concentrations of all ions, including the buffered ones. This section provides an example of how to compute total intracellular calcium concentrations, with buffer equations as in TTP06 and ORd CiPA. Usually, the models include buffers for Ca^{2+}, but any total ionic concentration can be computed following the example below. Total intracellular concentrations can be added as variables of the corresponding compartment. It is recommended to include them in the same compartment as the buffered concentrations, to make easier the eventual reading by other modellers.

Ten Tusscher 2006 model

In the TTP06 model, the free calcium concentration in the cytosolic space is updated taking into account a buffer:

$$\frac{\text{d}Ca_{i,free}}{\text{d}t} = BCa_i\left(\sum_{currents} I \times \frac{C_\text{m}}{V_c F} + \sum_{transporters} T_{yy'}\frac{V_{y'}}{V_y}\right),$$

with:

$$BCa_i = \frac{1}{1 + Buf_c\frac{K_{bufc}}{(Ca_i+K_{bufc})^2}}.$$

BCa_i is the fraction of free Ca^{2+}, K_{buf_c} is the equilibrium constant between the buffered Ca^{2+} and the free Ca^{2+}, and Buf_c is the concentration of buffer.

The expression of the buffer could be integrated so that:

$$Ca_{i,total} = Ca_i + Ca_i \frac{Buf_c}{Ca_i + K_{bufc}},$$

and

$$\frac{\mathrm{d}Ca_{i,total}}{\mathrm{d}t} = \sum_{currents} I \times \frac{C_{\mathrm{m}}}{V_c F} + \sum_{transporters} T_{yy'} \frac{V_{y'}}{V_y}.$$

The total concentration of calcium in the subspace and the SR are computed similarly for TTP06:

$$Ca_{ss,total} = Ca_{ss} + Ca_{ss} \frac{Buf_{ss}}{Ca_{ss} + K_{bufss}},$$

$$Ca_{sr,total} = Ca_{sr} + Ca_{sr} \frac{Buf_{sr}}{Ca_{sr} + K_{bufsr}}.$$

O'Hara CiPA 2017 model

In ORd CiPA, the free calcium concentration is computed similarly to TTP06 model, however, there are two buffers this time:

$$BCa_i = \frac{1}{1 + \frac{cm_{max} k_{cm}}{(Ca_i + k_{cm})^2} + \frac{tr_{max} k_{tr}}{(Ca_i + k_{tr})^2}}$$

Like previously, the total concentrations of calcium in the different compartments are computed as follows:

$$Ca_{i,total} = Ca_{i,free} \times \left(1 + \frac{cm_{max}}{Ca_{i,free} + k_{cm}} + \frac{tr_{max}}{Ca_{i,free} + k_{tr}}\right),$$

$$Ca_{ss,total} = Ca_{ss,free} \times \left(1 + \frac{BSR_{max}}{Ca_{ss,free} + Km_{BSR}} + \frac{BSL_{max}}{Ca_{ss,free} + Km_{BSL}}\right),$$

$$Ca_{jsr,total} = Ca_{jsr,free} \times \left(1 + \frac{csqn_{max}}{Ca_{jsr,free} + k_{csqn}}\right).$$

S1.3.2 Computing Γ_0 from the initial conditions

Γ_0 is computed after integrating Eq. 1, meaning that the integration constant must be computed to use the algebraic voltage expression. This can be done at initial state by re-arranging Eq. 6.

For TTP06, using the previous notation:

$$\Gamma_0 = K_i + Na_i + 2Ca_{i,total} + 2Ca_{sr,total}\frac{V_{sr}}{V_c} + 2Ca_{ss,total}\frac{V_{ss}}{V_c} - Na_{\mathrm{ext}} - K_{\mathrm{ext}} - Ca_{\mathrm{ext}} - \frac{VC_m}{FV_c}.$$

For ORd CiPA :

$$\Gamma_0 = K_i + Na_i + 2Ca_{i,total} + \left(K_{ss} + Na_{ss} + 2Ca_{ss,total}\right)\frac{V_{ss}}{V_c} + 2Ca_{sr,total}\frac{V_{sr}}{V_c} - Na_{\mathrm{ext}} - K_{\mathrm{ext}} - Ca_{\mathrm{ext}} - \frac{VC_m}{FV_c}.$$

S1.3.3 Swapping the voltage expression in your model

Before updating the expression for voltage in your model, it is advised to check that the difference between intra- and extracellular concentrations follows the voltage. It is recommended to run the model with the derivative voltage expression, and plot the voltage and the difference between intra- and extracellular concentrations, namely the part of Eq. 6 in brackets. The two curves should overlay, with a rescaling factor and offset. If not, this gives a hint about which concentration was omitted in the computation of total ionic intracellular concentrations.

To illustrate with TTP06 model, the voltage expression becomes:

$$V = \frac{FV_c}{C_m}\left(K_i + Na_i + 2Ca_{i,total} + 2Ca_{sr,total}\frac{V_{sr}}{V_c} + 2Ca_{ss,total}\frac{V_{ss}}{V_c} - Na_{\text{ext}} - K_{\text{ext}} - 2Ca_{\text{ext}} - \Gamma_0\right).$$

And the equation of voltage for ORd CiPA becomes:

$$V = \frac{FV_c}{C_m}\left(K_i + Na_i + 2Ca_{i,total} + (K_{ss} + Na_{ss} + 2Ca_{ss,total})\frac{V_{ss}}{V_c} + 2Ca_{sr,total}\frac{V_{sr}}{V_c} - Na_{\text{ext}} - K_{\text{ext}} - 2Ca_{\text{ext}} -\right.$$

S1.3.4 Troubleshooting

When trying to use the algebraic expression for voltage in a model provided with its derivative expression, several problems can arise. The list below points out eventual sources of “bugs” that were encountered during the present work and that could lead to a mismatch between the two voltage expressions:

- When using an user-defined value of Γ_0 for simulations, the voltage will be computed according to Eq. 6. However, due to the prefactors, small changes in Γ_0 lead to big differences in voltage, with approximately 0.01 mM changes in Γ_0 leading to 100 mV variations. Therefore, when changing the value of Γ_0, we recommend to adjust the initial concentration of potassium $[K^+]_i$ to maintain the initial voltage at physiological values. Otherwise, voltage can be pushed to values of several kV in extreme cases. The advantage of adjusting $[K^+]_i$ is that its range is high enough to enable a wide scan of Γ_0 values, while remaining positive.
- Note that the total membrane capacitance is computed differently from one model to another. It is usually introduced either as a model parameter, or as a product of capacitance per area unit and cell surface, or sometimes as a combination of capacitance per area unit, surface to volume ratio, and volume of the cell.
- The model must satisfy the conservation of charge principle. All the currents (including the stimulus current!) carry charges; all fluxes result in changes in ionic concentrations that should be included.
- If adjustment is needed for the model to satisfy the conservation of charge principle, check that the right currents are used to update the right concentrations. For example, Grandi 2010 model includes only the intracellular potassium concentration in the bulk cytosol, clamped to its initial value. The $Na^+ - K^+$ exchanger currents, however, are computed in the sarcolemma and junctional domains. This means that in sarcolemma and junctional domains, 3 Na^+ ions are exchanged for 2 K^+ ions, which should be accounted for in there. Thus, when considering dynamic potassium concentrations in the Grandi 2010 model, one needs to add variables for the K^+ concentrations in the different compartments of the model.
- Verify that **all of the charge-carrying species** were included in the voltage equation. One can check that by derivating over time the algebraic expression of voltage and comparing it with the sum of the

ionic currents through the membrane (derivative voltage expression). When derivating, do not forget that intracellular concentrations are not constant (important for buffered species).

- Verify that **all of the buffered forms** of the ions are taken into account for total ionic concentrations computation.
- The value for Γ_0 must be computed using the initial conditions for **total** ionic concentrations. This is particularly relevant for Ca^{2+} ions which are often buffered.
- The variable for voltage must be demoted from “state variable” to “computed variable”. Depending on the software/language used to solve the model, this might lead to problems.

S1.4 Convergence towards paced limit cycle

For this section, the absolute and the relative solver tolerances are set to the same value, ranging from 10^{-9} to 10^{-5}. TTP06 and ORd CiPA models are both used to simulate 3000 paces. The intracellular potassium concentration at the end of the AP (at time $t = 0ms$) is recorded and plotted for the extreme solver tolerances in Figures S1 to S4. Overall, the potassium concentrations converge towards the same value, no matter the solver tolerance used. The zooms on the phase of the simulation after the 2000th beat show however that the models do not always converge as the higher level view might suggest.

For high solver tolerances, the derivative voltage model is not even converging towards a paced limit cycle. Indeed, because of numerical error, the conservation of charge is not preserved anymore and there is divergence of the intracellular concentrations. On the other hand, the algebraic voltage model does converge, even though the reached value is visibly noisy around the limit cycle value. Note that the two models are mathematically identical, meaning that the differences in observed behaviours are only due to the numerical solving.

For low solver tolerances, both models seem to converge nicely to the same limit cycle value. The zoom on the “converged” phase shows that the derivative voltage model actually still drifts away, even with solver tolerances set to 10^{-9}. The slope of the drift is reducing with the fine solver tolerance though. In practice, this deviation would not induce any visible variations of the outputs usually studied. Still, this supports

To grade the stability of the model, a score taking into account the noisy variations around limit cycle values and the drifting away of $[K^+]_i$ was needed. From the higher scale view, it seemed that 2000 beats was a good cutoff to observe deviation from the limit cycle. Therefore, the 2000th beat was selected as reference, and the stability compared to that value. To quantify the deviation, the averaged distance to the reference value seemed to be indicative enough. To have a more visual map representing all together the various scales of deviation, a log-rescale was applied on the score..

S1.4.1 Comparison of solving speed between derivative and algebraic voltage expressions

The relative simulation time of solving the underlying sytem equations with the different voltage expressions were also compared, and similar maps are plotted in Figure S5 for the TTP06 and ORd CiPA models. These maps are mostly smooth, with the possible exception of ORd CiPA written with the algebraic voltage. As expected, increasing the solver tolerance increases the time-steps hence the speed of solving of the model. Also, using the algebraic voltage expression switches the voltage from a state variable to a computed variable. Having one variable less to compute should enable acceleration of the solving by CVODE. However, the maps of speed of solving do not agree with these expectations. Actually, as the same system can be described with the two expressions for voltage, this means that the system is overdetermined when written with the voltage as state variable. Therefore, writing the voltage as a computed variable breaks the overdetermination of the system. However, the time-step needed to meet with the solver tolerance does not change, and neither does the speed of solving. The break of the overdetermination of the system of equations describing the model could explain the improvement in simulation stability observed above though.

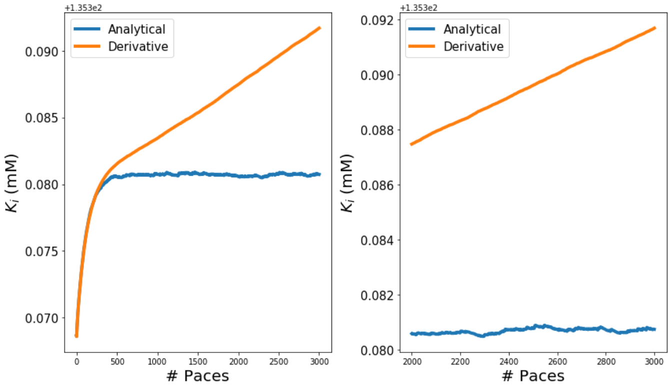

Figure S1. Convergence of intracellular potassium to its limit cycle value, for TTP06 model, for solver tolerances set to 10^{-5} . The **left** panel shows the convergence towards the paced limit cycle from the initial conditions. The **right** panel shows a zom on the evolution of the intracellular potassium after the 2000th beat where the model is supposed to have already reached the paced limit cycle.

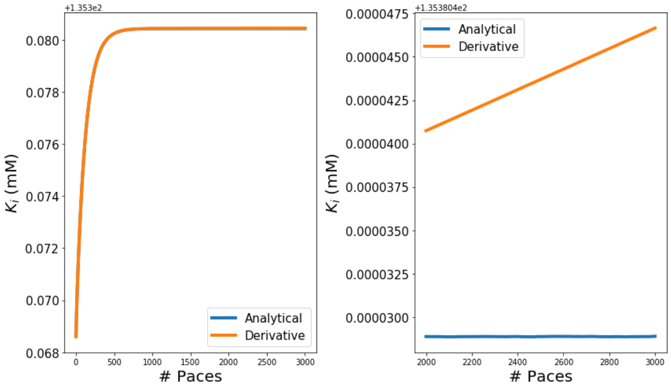

Figure S2. Convergence of intracellular potassium to its limit cycle value, for TTP06 model, for solver tolerances set to 10^{-9} . The **left** panel shows the convergence towards the paced limit cycle from the initial conditions. The **right** panel shows the evolution of the intracellular potassium after the 2000th beat where the model is supposed to have already reached the paced limit cycle.

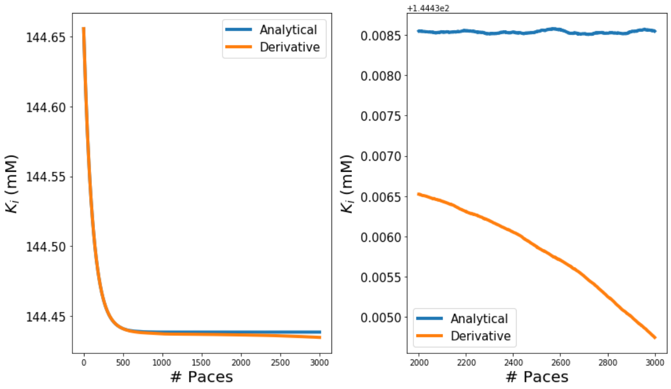

Figure S3. Convergence of intracellular potassium to its limit cycle value, for ORd CiPA model, for solver tolerances set to 10^{-5} . The **left** panel shows the convergence towards the paced limit cycle from the initial conditions. The **right** panel shows a zom on the evolution of the intracellular potassium after the 2000th beat where the model is supposed to have already reached the paced limit cycle.

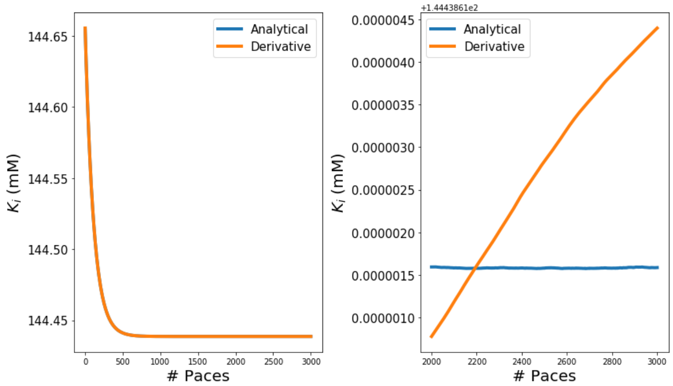

Figure S4. Convergence of intracellular potassium to its limit cycle value, for ORd CiPA model, for solver tolerances set to 10^{-9} . The **left** panel shows the convergence towards the paced limit cycle from the initial conditions. The **right** panel shows the evolution of the intracellular potassium after the 2000th beat where the model is supposed to have already reached the paced limit cycle.

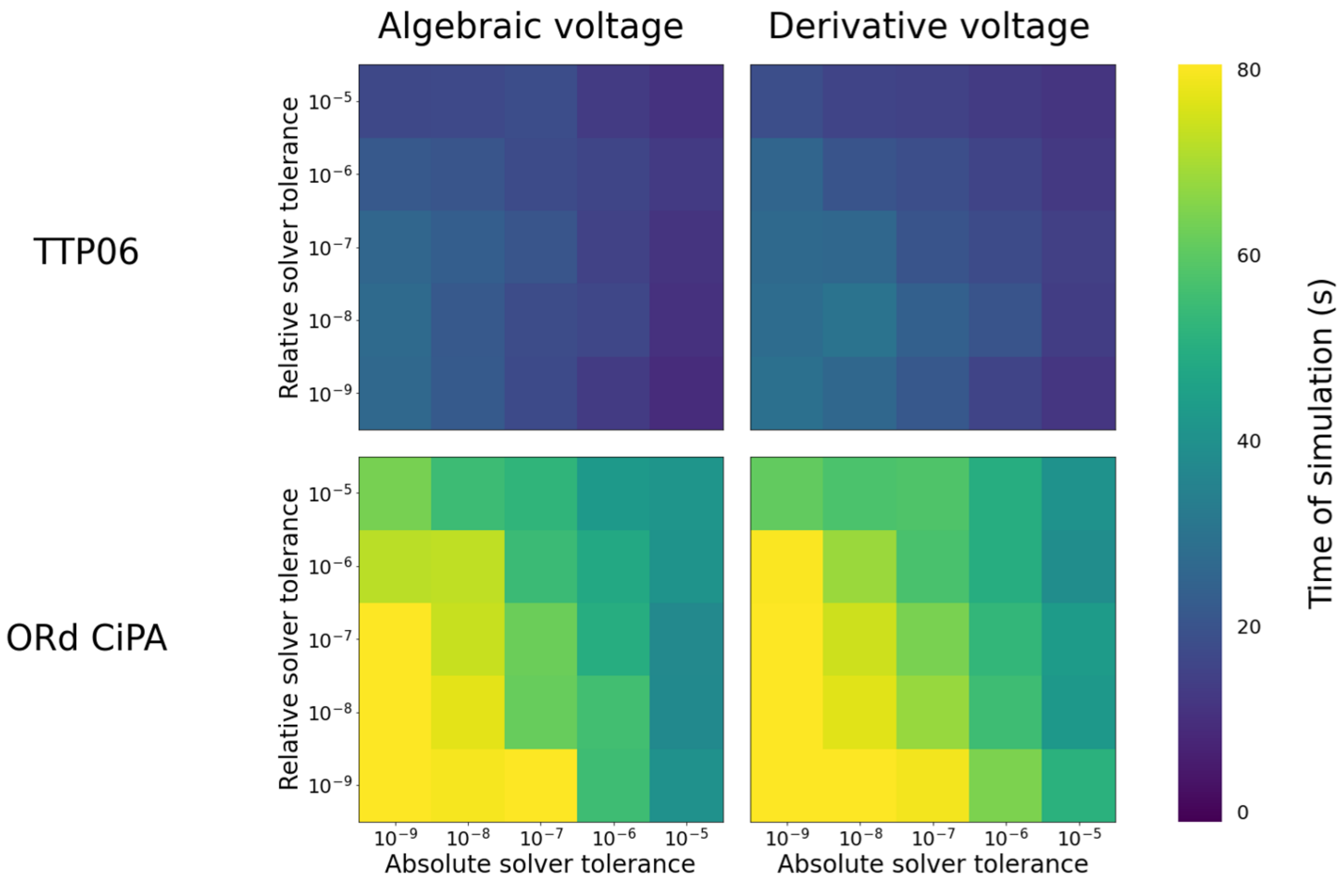

Figure S5. Map of speed for **A:** TTP06 model and **B:** ORd CiPA model, **Left:** using the algebraic voltage expression, and **Right:** using the derivative voltage expression. The colorcode corresponds to the speed of solving of the model. The darker the map, the lower the simulation duration, hence the faster the simulation the simulation.

REFERENCES

Endresen, L., Hall, K., Høye, J., and Myrheim, J. (2000). A theory for the membrane potential of living cells. *European Biophysics Journal* 29, 90–103. doi:10.1007/s002490050254

Hund, T., Kucera, J., Otani, N., and Rudy, Y. (2001). Ionic charge conservation and long-term steady state in the Luo–Rudy dynamic cell model. *Biophysical Journal* 81, 3324–3331. doi:10.1016/S0006-3495(01)75965-6

Jacquemet, V. (2007). Steady-state solutions in mathematical models of atrial cell electrophysiology and their stability. *Mathematical Biosciences* 208, 241–269. doi:10.1016/j.mbs.2006.10.007

Livshitz, L. and Rudy, Y. (2009). Uniqueness and stability of action potential models during rest, pacing, and conduction using problem-solving environment. *Biophysical Journal* 97, 1265–1276. doi:10.1016/j.bpj.2009.05.062

Ten Tusscher, K. and Panfilov, A. (2006). Alternans and spiral breakup in a human ventricular tissue model. *American Journal of Physiology: Heart and Circulatory Physiology* 291, H1088–H1100. doi:10.1152/ajpheart.00109.2006

Varghese, A. and Sell, G. (1997). A conservation principle and its effect on the formulation of Na–Ca exchanger current in cardiac cells. *Journal of theoretical Biology* 189, 33–40. doi:10.1006/jtbi.1997.0487

Yu, T., Lloyd, C., Nickerson, D., Cooling, M., Miller, A., Garny, A., et al. (2011). The Physiome Model Repository 2. *Bioinformatics* 27, 743–744. doi:10.1093/bioinformatics/btq723
